# Automatic air volume control system for ventilation of two patients using a single ventilator: a large animal model study

**DOI:** 10.1038/s41598-022-26922-4

**Published:** 2022-12-30

**Authors:** Krzysztof Zieliński, Barbara Lisowska, Katarzyna Siewruk, Maria Sady, Karolina Ferenc, Maciej Barwijuk, Jarosław Olszewski, Krzysztof Anusz, Artur Jabłoński, Magdalena Gajewska, Piotr Okrzeja, Marcin Michnikowski, Dorota G. Pijanowska, Krzysztof Pluta, Elżbieta Remiszewska, Marek Darowski, Romuald Zabielski, Adam Liebert, Katarzyna Kramek-Romanowska, Anna Stecka, Maciej Kozarski, Raman Pasledni, Zdzisław Gajewski, Piotr Ładyżyński

**Affiliations:** 1 Nalecz Institute of Biocybernetics and Biomedical Engineering, Polish Academy of Sciences, 4 Ks. Trojdena Str. 02109, Warsaw, Poland; 2 Department of Anesthesiology and Intensive Medical Care, National Geriatrics, Rheumatology and Rehabilitation Institute, Warsaw, Poland; 3 Veterinary Research Center, Center for Biomedical Research and Research Center for Regenerative Medicine, Warsaw University of Life Sciences – SGGW, Warsaw, Poland; 4 I Department of Anesthesiology and Intensive Care, Medical University of Warsaw, Warsaw, Poland; 5 Medical University of Warsaw, Warsaw, Poland; 6 Faculty of Chemical and Process Engineering, Warsaw University of Technology, Warsaw, Poland; 7 Center of Translational Medicine, Warsaw University of Life Sciences – SGGW, Warsaw, Poland

**Keywords:** Biomedical engineering, Respiration, Respiratory tract diseases

## Abstract

The COVID-19 pandemic outbreak led to a global ventilator shortage. Hence, various strategies for using a single ventilator to support multiple patients have been considered. A device called Ventil previously validated for independent lung ventilation was used in this study to evaluate its usability for shared ventilation. We performed experiments with a total number of 16 animals. Eight pairs of pigs were ventilated by a ventilator or anesthetic machine and by Ventil for up to 27 h. In one experiment, 200 ml of saline was introduced to one subject’s lungs to reduce their compliance. The experiments were analyzed in terms of arterial blood gases and respiratory parameters. In addition to the animal study, we performed a series of laboratory experiments with artificial lungs (ALs). The resistance and compliance of one AL (affected) were altered, while the tidal volume (TV) and peak pressure (Ppeak) in the second (unaffected) AL were analyzed. In addition, to assess the risk of transmission of pathogens between AL respiratory tracts, laboratory tests were performed using phantoms of virus particles. The physiological level of analyzed parameters in ventilated animals was maintained, except for CO_2_ tension, for which a permissive hypercapnia was indicated. Experiments did not lead to injuries in the animal’s lungs except for one subject, as indicated by CT scan analysis. In laboratory experiments, changes in TV and Ppeak in the unaffected AL were less than 11%, except for 2 cases where the TV change was 20%. No cross-contamination was found in simulations of pathogen transmission. We conclude that ventilation using Ventil can be considered safe in patients undergoing deep sedation without spontaneous breathing efforts.

## Introduction

In 2020, clinical centers around the world struggled with a shortage of ventilators due to the outbreak of *COVID-19*^1,2^. In many cases, *COVID-19* patients exhibit an atypical acute respiratory distress syndrome (*ARDS*)^3^, which leads to the necessity of mechanical ventilation. Thus, the global-scale problem of ventilator shortages is related to the sudden increase in the number of patients requiring long-term ventilatory therapy that has exceeded the number of ventilators available.

In several reports published several years ago, anesthesiologists considered solving the problem of a lack of ventilators by sharing a single ventilator for simultaneous therapy of two or even up to four patients^4–6^. However, this approach has been criticized as insufficient to maintain stable ventilation for each patient in the event of changes in lung mechanics in any of the ventilated patients^7^. Following the COVID-19 outbreak, various new configurations of ventilator sharing systems have been proposed and tested^8–12^. These systems provide varying degrees of control over ventilatory branches, delivering airflow to the patients. Most of these solutions use manually titrated pneumatic valves. However, these solutions increase patient monitoring efforts and do not guarantee safe, multiple patient ventilation. The risk associated with such a ventilatory strategy is relatively high; however, it can be considered a potential bridge to full ventilatory support of the patient^4,11,13–18^.

Another concept of shared ventilation is based on continuous automatic control of the tidal volume division between ventilated subjects^27^. This technical approach was initially applied for independent lung ventilation and validated in a series of laboratory experiments^28,29^ and clinical tests carried out in patients ventilated during thoracic surgeries^30^ or undergoing differential ventilation therapy in an intensive care unit^31^. Based on these studies, we developed a medical device called Ventil that could allow the ventilation of two patients with one ventilator. Therefore, the main goal of this study was to validate the usability of Ventil in shared ventilation using an animal model. The aim of the animal experiments was to evaluate arterial blood gases and respiratory parameters of ventilated subjects with different weights by means of a ventilator/anesthetic machine and Ventil. The shared ventilation approach carries the risk of mechanical ventilation alterations in one patient when the respiratory mechanics in the second patient change (respiratory deterioration or improvement). Therefore, supplementarily to animal experiments, the laboratory investigations were performed using artificial lungs (AL). The aim of laboratory tests was to evaluate alterations in tidal volume and inspiratory peak pressure of an unaffected ventilated AL when the mechanical properties of the respiratory system (resistance and compliance) were altered in the second (affected) AL.

Another clinical problem of shared ventilation is the risk of transmission of pathogens in the respiratory tract. Then, a research problem arises, resulting from a lack of fully defined methods that can be used to assess the transport of pathogens in the ventilation duct under laboratory conditions in a short time and from not considering the risk of biohazards. For pathogens such as viruses, the term “viral load”, expressed as a titer, is a numerical representation of the quantity of virus in a given volume of fluid in a sample, e.g., saliva, blood, plasma, etc. For viruses transmitted via airborne droplets, the most common method for viral titer estimation is quantitative *PCR* (*qPCR*) performed on nasopharyngeal swab samples. Despite the large body of evidence concerning *SARS-CoV-2*, *SARS-CoV*, and *MERS-CoV* viral load dynamics, duration of viral shedding, and infectiousness^19^ published to date, there are few or no data on the virus load required for human infection. Nonetheless, Han et al. showed that the average titer of the initial viral load obtained from swab specimens taken from asymptomatic children was ca. 2 × 10^6^ genomic *RNA* copies per mL, whereas symptomatic children had an initial viral load as high as 1 × 10^9^^20^.

Moreover, according to Chu et al., in the culture medium of *Caco2* and *Calu3* cells infected with *SARS-CoV-2* virus, the number of virus genome copies 120 h after infection was determined to be 10^9^ in 1 mL of supernatant^21^. The multiplicity of infection (*MOI*) was 0.1. In our studies using the viral phantom in the form of fluorescently labeled nanospheres, the estimated phantom titer was determined from an exponential pattern of viral reproduction in the respiratory system. Another published study showed that the minimal dose of *MERS-CoV* required to effectively infect a cell culture was 10^7^ copies per mL^22^. In the case of *SARS-CoV-2*, no infection occurred with a titer below 10^6^ copies per mL^23^, cycle threshold values higher than 24 in another study^24^ or cycle threshold values higher than 34 in other studies^25,26^. Thus, laboratory tests of pathogen transmission were performed. For the investigation of pathogen spread along the respiratory tract and contamination of the second tract, fluorescent nanospheres were used as a phantom of virus particles.

## Methods

### Ventil device

**Figure 1:**
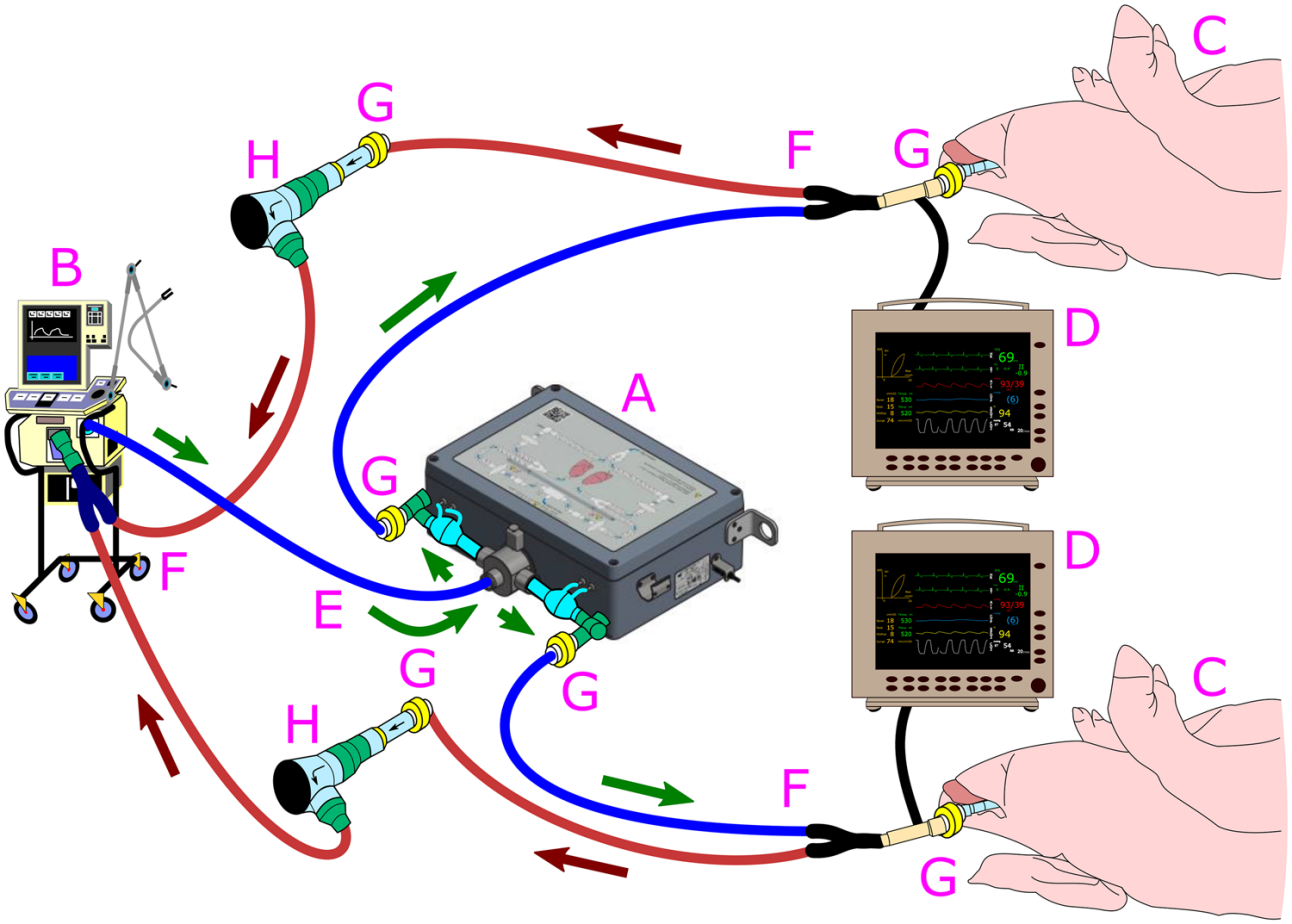
Ventilation of two pigs was performed with a Ventil and a ventilator. Animal experiment test stand. A, Ventil; B, ventilator or anesthetic machine; C, ventilated subject; D, patient monitor; E, adult polypropylene extendible limb 22F-22F; F, adult polypropylene anesthesia extendible breathing circuits with Y-pieces; G, antibacterial and antiviral filter; H, positive end-expiratory valve with one-way valve and set of connectors; green arrows—the flow direction during the inspiration; dark red arrows—the flow direction during the expiration.

Ventil is a device initially developed for differential mechanical lung ventilation and can be used in one-lung ventilation as well as in the case of asymmetric lung pathologies. The apparatus splits the flow according to the selected ratio (by a division knob). However, it must be emphasized that Ventil is not a simple splitter of the flow from the ventilator. Rather, it is a device that can adjust minute ventilation, keeping the parameters stable regardless of changes in the air duct properties of the lungs. The division of the flow is stabilized by two flowmeters, continuously measuring the flows in output ports and sending these flow signals to the control system, which then corrects the splitting to maintain the flow division according to the selected ratio. External mechanical or electromagnetic (fixed or adjustable) positive end-expiratory pressure (*PEEP*) valves can be used with the Ventil. Therefore, the *PEEP* for both lungs can also be regulated. Ventil requires one-way valves to separate tracks (particular lung circuits) and protect them from gas mixing. The Ventil apparatus is placed in the breathing circuit between a ventilator and the ventilated subjects (as shown in Fig. 1 and S1). It does not communicate with the ventilator; thus, any kind of ventilating machine can be used without its modification. We manufactured a short series of 200 Ventil devices. Moreover, Ventil was certified for independent ventilation of two lungs in a single patient (manufacturer Łukasiewicz-ITAM, Zabrze, Poland). Technically, Ventil can be used to ventilate two patients instead of two lungs.

### The test stand during experiments on animals

Two Puritan Bennett 980 and 840 ventilators (Medtronic, Minneapolis, MN) and two Anastazja 7700 anesthesia ventilators (Farum Ltd., Warsaw, Poland) were used with two Ventil devices (serial numbers *SN0022* and *SN0024*). Four patient monitors (Datex-Ohmeda S/5, GE Healthcare, Boston, MA) were used with the E-PRESTN module (GE Healthcare, Boston, MA). Three of them were also equipped with an E-CAIOV module (GE Healthcare, Boston, MA). Data from patient monitors were collected by Windows 10 PC-based computers. We calculated the median and interquartile range (*IQR*) values for the following continuously recorded signals during the experiments: oxygen saturation (SpO_2_), oxygen inspiratory fraction (FiO_2_), end-tidal carbon dioxide partial pressure (EtCO_2_), peak inspiratory pressure (*PIP*), positive end-expiratory pressure (*PEEP)*, respiratory rate (*RR*), and tidal volume (*TV*) and calculated the following parameters: driving pressure (ΔP), minute ventilation (*MV*) and static compliance (*Cst*). The *MV* was an inspired tidal volume times *RR*. The driving pressure was calculated as the difference between the plateau pressure (*Pplat*) and the positive end-expiratory pressure (*Pplat* – *PEEP*). The static compliance was the ratio of the expired *TV* and the driving pressure. These 3 parameters were calculated per recorded sample. The statistics were processed for all pigs used in the experiments. The spirometric variables were not recorded by the fourth patient monitor due to the E-CAIO (GE Healthcare, Boston, MA) instead of the E-CAIOV module implemented (the E-CAIO module does not measure spirometric variables, in contrast to the E-CAIOV module). All recorded data were postprocessed by MATLAB R2019b and MS Excel 2019 software.

Two Ventil flowmeters (Ventil outputs) were connected with inspiratory arms of two adult polypropylene (PP) anesthesia extendible 60/180 cm breathing circuits with Y-pieces (Medtronic, Minneapolis, MN) by Fixed elbow 22 M—7.6 mm port—22 M/15F connectors (Intersurgical, New York, NY) and electrostatic antibacterial and antiviral Barrierbac S 22 M/15F filters (Medtronic, Minneapolis, MN) in series. The Y-pieces were connected to the animals by electrostatic filters (various vendors) and the patient-monitored probes. The animals' expiratory limps were connected to the electrostatic antibacterial and antiviral Barrierbac S 22 M/15F filters (Medtronic, Minneapolis, MN), one-way valves (Intersurgical, New York, NY), and 2.5 cmH_2_O *PEEP* valves (Intersurgical, New York, NY or Flexicare, Mountain Ash, Great Britain). The *PEEP* valve output was connected (through 30 M-22 M [Intersurgical, New York, NY] or 22 M-22 M/15F [R-Vent Medikal, İzmir, Turkey] connectors) by third adult PP anesthesia extensible 60/180 cm breathing circuits (Medtronic, Minneapolis, MN) to the ventilator (Y-piece site) by a 22F-22F connector (Intersurgical, New York, NY). The configuration is presented in Fig. 1.

### Animal use protocol

We investigated 16 pigs in 8 pairs ventilated by the Ventil and a ventilator or an anesthetic machine (Fig. 1) set to volume-controlled ventilation modes. This animal study was approved by the First Bioethical Commission in the Ethics Committee for Animal Experiments in Warsaw (agreement no WAW2/047/2020). All methods were carried out in accordance with relevant guidelines and regulations and reported in accordance with ARRIVE guidelines (https://arriveguidelines.org). After obtaining approval, the following animals (pigs) were classified for testing: males and females of the Great White Polish Breed, aged approximately 3–6 months. The choice of pigs was based on the assumption of the similarity of body and lung mass and therefore ventilation parameters to those of humans, with a tidal volume of 6–8 ml/kg. Preparation for qualified animals was carried out according to an established schedule. Prior to intubation and mechanical ventilation, the animals received premedication: medetomidine 0.05–0.1 mg/kg (Cepetor 1 mg/ml, ScanVet Ltd., Warsaw, Poland), butorfanol 0.1–0.2 mg/kg (Butomidor 10 ml, Richter Pharma AG, Wels, Austria), and ketamine 5–10 mg/kg (Bioketan, Vetoquinol Biowet Ltd., Gorzów Wielkopolski, Poland) as an intramuscular injection (buttock muscles). After securing an intravenous line (BD Venflon 1.2 mm, Becton Dickinson, Franklin Lakes, NJ) with access to the posterior ear vein (vena auricularis posteriori), the animals were induced into general anesthesia with propofol (Scanofol 10 mg/ml, ScanVet Ltd., Warsaw, Poland) at a dose based on the body weight (4.5 mg/kg). After intubation (the size of the endotracheal tubes was visually and manually determined based on the weight of the animals and was in the range of 6–11), general anesthesia was conducted with isoflurane (Aerrane Baxter Healthcare, Baxter Inc., Warsaw, Poland) in a volumetric percentage adequate to the induced clinical effect, starting with a concentration of 5 vol%, with a continuation of 2 vol%. Subsequently, the anesthetized animals underwent a computer tomography (CT) scan of the thorax (lungs were inflated with air) and then were connected to the anesthesia machine or ventilator by Ventil. All animals under this experiment had artery cannulas placed in the iliac artery and a central intravenous line in the iliac vein, and both were used to assess cardiovascular indices and to obtain blood samples for laboratory tests. For all subjects, a bladder catheter was also placed to assess diuresis. Anesthesia was conducted with isoflurane during the experiment when the animals were ventilated by an anesthetic machine, while propofol (2 mg/kg/h) was applied in those experiments when the ventilator was used. During all experiments, we adjusted the tidal volume, *RR*, maximal flow (*V̇max*), FiO_2_, inspiratory-to-expiratory ratio (*I:E*) and *PEEP* in the ventilator and the Ventil division knob to keep animal respiratory variables in the physiological range. We changed the position of the ventilated animals at least every 4 h. Arterial blood gas (*ABG*) samples were collected and analyzed by a blood analyzer (epoc® Blood Analysis System, Siemens Healthineers, Erlangen, Germany) in terms of the activity of hydrogen ions (*pH*), carbon dioxide partial pressure (pCO_2_), oxygen partial pressure (pO_2_), bicarbonate concentration (HCO_3_^−^), base excess in the extracellular fluid (*BE (ecf)*), oxygen saturation of hemoglobin (SaO_2_) and the lactate level.

In all experiments except No. 3, *ABG* samples were collected at 0, 5, 12, 17, and 24 h of the experiment from all subjects. In these experiments, we ventilated the animals for at least 24 h. In experiment No. 3, *ABG* was recorded at 0, 2, 4, 6, 8, and 10 h, and this experiment was run for 11 h. In this experiment, in the 3rd and 8th hours, we introduced 100 ml of bronchopulmonary saline (in a total of 200 ml) to pig #1 to reduce lung compliance (as in *ARDS*). The purpose of this assessment was to assess the influence of deterioration of mechanical properties of the lungs/respiratory track in one subject on the ventilatory variables of the second subject.

After the experiment, a CT scan of the thorax was repeated for all animals. At the end of the experiment, when the first ventilated animal was undergoing a CT scan , a 2 L respiratory bag was used instead. In experiment no 3, CT scans were additionally performed during ventilation. When one animal was undergoing a CT scan, a 2 L respiratory bag was used instead while the experiment continued. We did not analyze these additional CT scans in this study. We investigated all *CT* scans at the beginning and end of the experiments in terms of the presence of emphysema, pneumothorax and pleural effusion.

### Statistical analysis

The pairs of pigs were divided into two groups in terms of weight: the WS group included pigs with equal or similar weights, and the WD group included pigs with different weights. We examined arterial blood gas parameters. The Mann–Whitney U test was used to compare the differences between all values of *ABG* parameters in pairs with WS and WD groups acquired across the experiments because the distribution of these parameters differed from the normal distribution and because for most of them, the homogeneity of variances was not fulfilled. For all experiments except no. 3, the Friedman test and Spearman's rank correlation were performed to analyze the repeatable *AGB* parameters measured at fixed intervals of time (at 0, 5, 12, 17, and 24 h). The analyses were performed separately for each of the parameters for the *WS* and *WD* groups. The value for a statistically significant difference was set at α = 0.05 for all statistical analyses. The Statistica v.13.3 software package was used for the calculations.

### Laboratory test bench

A Ventil with serial number *SN0003* equipped with two flowmeters (SpiroQuant H, EnviteC, Germany) was used. Two elbow connectors with luer-lock ports (22 M/15F- and straight connectors (22 M-22 M and 22F-22F) were connected the Ventil flowmeters and the air-gas flowmeters SFM3000 (Sensirion, Switzerland). Two adult PP anesthesia extendible 60/180 cm breathing circuits with Y-pieces (Medtronic, Minneapolis, MN) were connected to SFM 3000 (inspiratory arms) with artificial test lungs SmartLung 2000 (IMT Analytics, Switzerland) through HMEF filters. Expiratory limbs were connected to electrostatic antibacterial and antiviral filters Barrierbac S 22 M/15F (Medtronic, Minneapolis, MN), one-way valves (Intersurgical, New York, NY), and 2.5 cmH_2_O *PEEP* valves (Intersurgical, New York, NY). The *PEEP* valve output was connected (through 30 M-22 M connectors (Intersurgical, New York, NY)) by third adult PP anesthesia extensible 60/108 cm breathing circuits (Medtronic, Minneapolis, MN) to the ventilator (Y-piece site) by a 22F-22F connector (Intersurgical, New York, NY). Luer-lock ports were connected with 143SC01D-PCB pressure sensors (Sensortechnics GmbH, Germany). These pressure sensors and SFM3000 flow sensors are part of the measurement system. Pressure signals were recorded by the real-time NI PXI-1042 system with an NI PXI-6289 data acquisition board installed (both of them from National Instruments, Austin, TX). Flow data from SFM3000 flow sensors were recorded by the STM32VLDISCOVERY board (STMicroelectronics, France-Italy). All pressure and flow data were collected by a Windows 7 PC laptop with its own developed software in LabVIEW™ 2013 (National Instruments, Austin, TX) for data storage and visualization. The configuration is presented in Fig. S1. The picture with the given setup is shown in Fig. S2.

### Laboratory experiments: effects of changing resistance and compliance in one artificial lung (*AL*) on flow and pressure in the second lung

We changed the resistance (*R*, all values expressed in mbar/L/s) from the baseline value *R* = 5 to values 20 (*R5 to R20*), 50 (*R5 to R50*) and 200 (*R5 to R200*) and compliance (*C*, all values expressed in mL/mbar) from baseline value *C* = 75 to values 60 (*C75 to C60*) and 25 (*C75 to C25*) as well as from *C* = 60 (for both *AL*) to *C* = 75 (C60 to C75). We also performed breathing circuit disconnection (in the patient's filter point – *P1* marker in Fig. S1) tests (*R5 to R0*) and replaced the first *AL* (*R* = 5, *C* = 75) with the 2-L respiratory bag (Medtronic, Minneapolis, MN) with *R* ~ 0 and *C* ~ 15 (*AL to Bag*). The second *AL* parameters were not affected during the experiments. For the ‘R5 to R20’, ‘R5 to R50’, ‘R5 to R200’ and ‘R5 to R0’ events, a baseline C = 75 value was used. For the ‘C75 to C60’, ‘C75 to C25’ and ‘C60 to C75’ events, a baseline *R* = 5 value was used. Baseline *R* = 5 and *C* = 75 values were used for *AL* in the ‘AL to Bag’ event. We investigated how the pressure and delivered tidal volume were changed in AL *#2* (expressed by index 2 for pressures and volumes in Table 4) when the parameters of AL *#1* were changed (pressures and volumes in Table 4 for this *AL* are expressed by index 1). All tests were performed for three respiratory rate values of 12, 18, and 24 breaths/min.

### Simulation of cross-contamination

The experimental measurement system and conditions are described in supplement R1—Technical Report of IBBE PAS on the transmission of nanoparticles/solutions in two respiratory branches of the Ventil system for experiments carried out in the period April 4–29, 2020. Figure R1 (technical report R1) shows a diagram of the test system with the Ventil apparatus supplying the respiratory tract during independent ventilation of two artificial lungs (right and left). Left: Right flow ratio 6:4. To test the possibility of transmission between the two airways, the test solutions and phantom suspensions were administered to the expiratory tract of the artificial *L* lung using an Areogen nebulizer (*N*) in 3 mL portions repeated several times. The checkpoints for the presence of test fluorescent substances and nanoparticles were at test points *T1*, *T2*, and *T3*. In the experiments, fluorescent compounds, such as sodium fluorescein (fluorescein) and methylene blue, purchased from Sigma and fluorescent red and green polystyrene nanospheres (red fluorescent polystyrene microspheres—EPRUI-RF-100C and green fluorescent polystyrene microspheres—EPRUI-GF-100C) with a diameter of 100 nm were used as virus phantoms. The stable fluorescence of the nanospheres was ensured by the incorporation of the dye inside them (so-called internal labeling). Fluorescein and methylene blue solutions at a concentration of 1 mM were used. In contrast, fluorescent nanospheres were administered as a 60 µL suspension and 240 µL stock suspension (provided by the manufacturer) per 3 mL of deionized water. Then, the estimated number of nanospheres in the prepared suspensions was 6.5.1012 and 24.1012 particles per 3 mL, respectively.

## Results

### Animal experiments

Of 8 experiments, one was abandoned after 4 h due to decreasing saturation and cardiorespiratory disorders in both animals. Asystole was found in one animal along with no response to administered drugs during resuscitation. In addition, dysfunction of unidirectional mechanical positive end-expiratory pressure (*PEEP*) components in the circuit was found, possibly causing mixing of exhaled gases in the respiratory circuits of the ventilated subjects. Another experiment was aborted after approximately 8–9 h due to carbon dioxide accumulation, and the body temperature increased up to 43 °C in one pig. The animal died as a result of asystole. Malignant hyperthermia was considered the cause of death. These experiments were excluded from the analyses. The configuration of the other 6 successfully completed experiments is summarized in Tables 1 and 2.

**Table 1:** Animal experiment configuration.

Pair no	Weight (kg) #1/#2	Sex #1/#2	Device	Rationale	Ventilation time (h)	Group
1	92/82	M/M	Anastazja 7700 + Ventil	Objects with similar weights ventilated by an anesthetic machine	27	WS
2	80/76	F/F	Bennett 980 + Ventil	Objects with similar weights ventilated by a ventilator	24	WS
3	62/61	F/F	Bennett 980 + Ventil	Objects with similar weights ventilated by a ventilator (compliance reducing)	11	WS
4	31/68	F/F	Bennett 840 + Ventil	Objects with different weights ventilated by a ventilator	24	WD
5	35/74	F/F	Bennett 980 + Ventil	Objects with different weights ventilated by a ventilator	24	WD
6	94/65	F/F	Bennett 980 + Ventil	Objects with different weights ventilated by a ventilator	24	WD

Ventilation of different animal pairs by the Ventil together with the anesthetic machine or the ventilator.

^#^1, The first ventilated subject; #2, The second ventilated subject; WS, Similar weight group; WD, Different weight group.

**Table 2:** Animal experiments results.

Pair no		1		2		3		4		5		6	
Pig no		#1	#2	#1	#2	#1	#2	#1	#2	#1	#2	#1	#2
Variable	Unit	Median (Iqr)	Median (Iqr)	Median (Iqr)	Median (Iqr)	Median (Iqr)	Median (Iqr)	Median (Iqr)	Median (Iqr)	Median (Iqr)	Median (Iqr)	Median (Iqr)	Median (Iqr)
TV	ml/kg	4.15 (4.49 - 3.73)	4.98 (5.39 - 4.37)	4.96 (5.19 - 4.80)	4.88 (5.08 - 4.75)	7.17 (7.29 - 7.06)	7.05 (7.20 - 6.93)	-	5.58 (5.68 - 5.51)	6.79 (7.14 - 6.40)	5.52 (5.78 - 5.08)	5.83 (6.04 - 5.70)	6.96 (7.09 - 6.82)
RR	1/min	28 (28 - 26)	28 (28 - 25)	24 (24 - 24)	24 (24 - 24)	22 (24 - 22)	24 (24 - 22)	25** (26 - 25)	25 (26 - 25)	27 (27 - 26)	27 (27 - 26)	21 (22 - 20)	21 (22 - 20)
FiO2	–	0.44 (0.52 - 0.42)	0.44 (0.51 - 0.42)	0.40 (0.40 - 0.35)	0.40 (0.40 - 0.35)	0.53 (0.54 - 0.44)	0.54 (0.54 - 0.44)	0.29** (0.44 - 0.29)	0.29 (0.44 - 0.29)	0.48 (0.61 - 0.44)	0.49 (0.51 - 0.44)	0.35 (0.45 - 0.34)	0.34 (0.44 - 0.33)
SpO2	%	96 (97 - 94.7)	96 (97.5 - 95.5)	95 (96.8 - 90.4)	94 (95.4 - 91.9)	96 (98.1 - 94.8)	100 (100 - 97.5)	100 (100 - 99.4)	95 (96 - 94.3)	97 (98.2 - 96.8)	94 (96.7 - 92.1)	95 (96.2 - 93.5)	94 (95.2 - 93.6)
EtCO2	mmHg	53.2 (61 - 49.7)	65.6 (81 - 55.9)	54.4 (57 - 52.3)	45.4 (49.8 - 43.4)	51.5 (53.7 - 49.3)	47.8 (49 - 46.6)	-	49.0 (50.6 - 48)	50.2 (52.1 - 46.7)	42.6 (45.2 - 39.3)	52.9 (54.7 - 49.7)	49.9 (57.2 - 48.5)
PIP	cmH_2_O	17.4 (19.4 - 15.8)	17.60 (18.9 - 16.3)	17.9 (18.3 - 17.3)	18.9 (19.6 - 17.7)	24.0 (27 - 20.9)	21.0 (22.4 - 18.7)	-	17.6 (18.1 - 17.1)	29.1 (33.1 - 26)	20.1 (21 - 18.9)	18.4 (18.9 - 17.9)	18.5 (18.8 - 18.1)
PEEPi	cmH_2_O	12.1 (13.1 - 8.6)	12.6 (13.2 - 11.6)	12.2 (13.2 - 11.3)	12.1 (12.9 - 11.3)	10 (10.6 - 9.4)	9 (9.7 - 8.4)	-	9 (9.7 - 8.7)	11.5 (12.5 - 9.6)	13.3 (13.8 - 12.6)	8.9 (10 - 8.2)	9 (9.7 - 8.3)
ΔP	cmH_2_O	4.1 (8.4 - − 3.1)*	4.2 (7.2 - − 1.4)*	2.5 (3.4 - 1.6)	3.3 (4.4 - 1.6)	12 (13.7 - 9.2)	8.3 (9.3 - 7.4)	-	6.1 (6.7 - 5.4)	10.6 (14.5 - 9.5)	3.7 (4.3 - 3)	6.8 (7.4 - 6.2)	7 (7.7 - 6.4)
Cst	ml/cmH_2_O	36.07 (56.3 - − 62.1)*	48.50 (86.4 - − 51.9)*	143.52 (196 - 86.9)	89.65 (116.9 - 44.5)	37.88 (49.2 - 33.1)	52.11 (57.4 - 47.3)	-	60.77 (67.1 - 55.5)	25.27 (28 - 16.6)	108.53 (136.2 - 93.2)	81.14 (90.6 - 73.5)	63.85 (69.3 - 59.3)

Median and interquartile range values of selected ventilatory parameters for the ventilated pairs.

TV, Tidal volume; RR, Respiratory rate; FiO_2_, Oxygen fraction in the inhaled gas; SpO_2_, Arterial blood saturation; EtCO_2_, End-tidal carbon dioxide; PIP Peak inspiratory pressure; PEEPi, Intrinsic positive end-expiratory pressure; ΔP, Driving pressure; Cst, Static lung compliance; IQR, Interquartile range.

*The negative driving pressure and static compliance are due to highly noisy signals for pair no. 1.

**Because the spirometric variables were not collected for object #1, the median and IQR values of FiO_2_ and RR were copied from the object #2, as the two objects had these values in common.

The median and lower IQR bound of SpO_2_ for all animals were higher than the clinically accepted level of 90%. The median inspiratory oxygen fractions, in most cases, were below 0.5. The median EtCO_2_ level was above the range considered normal (35–45 mmHg). The level of permissive hypercapnia in the ventilated subjects was up to 65.6 mmHg (median EtCO_2_ values during the experiments). The median driving pressure was below 10 cmH_2_O for all animals except 2 cases: Experiment 5, pig *#1* with chronic emphysema and pneumothorax of the left lung and pig *#1* in Experiment 3 with reduced compliance. These two pigs are characterized by lower (median) static lung compliance than the others. They also have the highest *PIP* values. The median *TV* (expressed in ml per kg) of 8 animals was in the range of 4–6 ml/kg recommended for mechanical ventilation in *ARDS* subjects, while in 3 animals, the *TV* was higher than 6 ml/kg up to 7.17 (in one animal, the *TV* was not measured).

### Computer tomography scan analyses

We did not find emphysema in any subject before and after ventilation, except subject *#1* (Experiment 5), in which right-sided emphysema was indicated before and after the experiment. We did not find pneumothorax in any subject before and after ventilation, except the same animal that presented signs of emphysema, in which left-sided pneumothorax was indicated before and both-sided pneumothorax after the experiment. We did not find pleural effusion in any subject before the experiment and only trace amounts on both sides in subject *#2* (Experiment 1) after ventilation.

The analysis of *CT* scans showed that, generally, Ventil together with a ventilator or anesthetic machines did not cause any severe lung injuries in ventilated animals, with one exception, namely, subject *#1* in Experiment 5, which was characterized by chronic emphysema and pneumothorax before the experiment and then very low lung compliance. This animal had the highest median *PIP* value and *ΔP* value over 10 cmH_2_O.

### Time-trend analysis of the selected animal experiments

**Figure 2:**
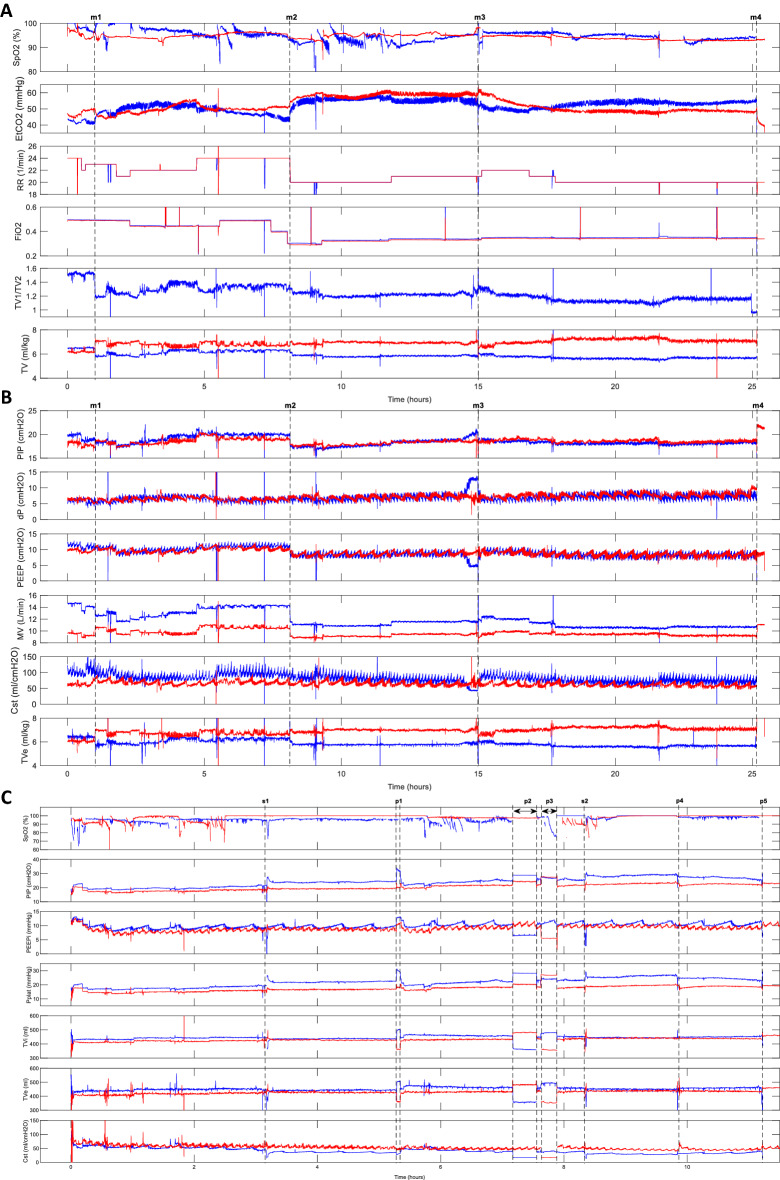
Time traces for the selected animal experiments. (**A**, **B**) Ventilatory parameters over time for Experiment (pair) 6. (**C**) Ventilatory parameters for Experiment 3. Blue traces denote object #1, while red traces denote object #2 (except *TV1/TV2*). Markers: m1, Changing the Ventil flow division knob; m2, Respiratory rate reduction; m3, Animal position change from the left side to the right side; m4, One object being exchanged with a 2 L respiratory bag; S1, Injection of the first 100 ml of saline to pig #1; p1, Flow division changes by the Ventil; p2 and p3, The times when a 2-L respiratory bag was connected instead of pig #1 and #2, respectively; s2, Injection of the second 100 ml of saline to pig #1; p4, Animal position change from the right side to the left side; p5, Exchange of the #1 object with a 2 L respiratory bag; MV, Minute ventilation; TV, Tidal volume; FiO_2_, Oxygen fraction in the inhaled gas; SpO_2_, Arterial blood saturation; EtCO_2_, End-tidal carbon dioxide; TV1/TV2, Tidal volume ratio—ventilation ratio; PIP, Peak inspiratory pressure; Pplat, Plateau pressure; PEEPi, Intrinsic positive end-expiratory pressure; ΔP, Driving pressure (Pplat–PEEPi); TVe, Expiratory tidal volume, Cst, Static lung compliance.

In Fig. 2A,B, we present the time trends of Experiment 6. In the time trends, we marked *m1*–*m4* symbols indicating exemplary events related to the experiment. Marker *m1* indicates the time when the Ventil flow division ratio was significantly changed to decrease EtCO_2_ in subject *#2* and increase it in subject *#1*. Marker *m2* indicates the time when *RR* and FiO_2_ were decreased. Marker *m3* indicates the time when the side on which the pigs were lying was changed. Before this, we found a rapid increase in *PIP* and then *ΔP* and a reduction in intrinsic *PEEP* and the static compliance *Cst* of subject *#1*. The body temperature of this pig tended to be distinctly elevated on the side it was lying on during all the experiments. This temperature elevation probably accompanied the *Cst* decrease, as these rapid *Cst* changes disappeared after the body position was changed. The rapid decrease in *Cst* on #1 did not cause significant changes in the ventilation of subject #2. Marker *m4* shows the end of the experiment and the moment pig #1 was disconnected, and a 2-L respiratory bag was terminated to the breathing circuit. This resulted in a tidal volume increase for pig #2, which was followed by an increase in *PIP* and a decrease in EtCO_2_. This phenomenon we also found in a second experiment is described in the next paragraph.

The time trends of experiment no. 3 are presented in Fig. 2C. Marker S1 indicates the injection of the first 100 ml of saline into the lungs of pig *#1*. It caused a gradual increase in *PIP* in pig *#1*, while in pig *#2*, the ventilatory parameters were not changed significantly. Marker *p1* indicates the time when the ventilatory parameters were changed. Markers *p2* and *p3* indicate the time periods when a 2-L respiratory bag was connected instead of pigs *#1* and *#2*, respectively. Marker S2 indicates the injection of the second 100 ml portion of saline into pig *#1*. This caused the next gradual increase in *PIP* in this subject. However, it did not affect the ventilation of pig *#2*. Marker *p4* is the time when the side on which the pigs were lying was changed from right to left. Marker *p5* indicates the disconnection of pig *#1* and the connection of the 2-L respiratory bag into the breathing circuit.

### Arterial blood gas analysis in animal experiments

**Figure 3:**
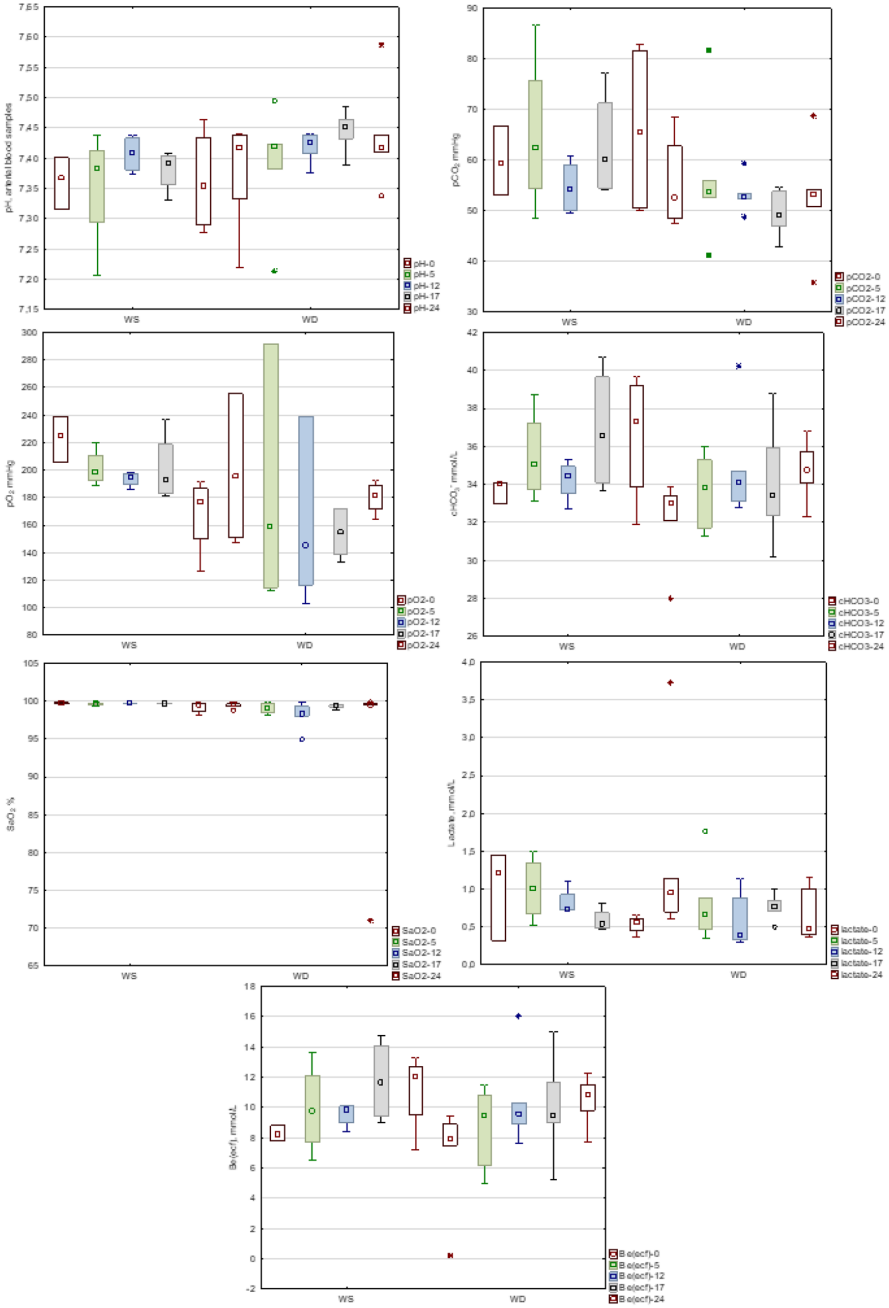
Arterial blood parameters were recorded at 0, 5, 12, 17 and 24 h in Experiments 1 and 2 (WS group) and Experiments 4, 5, and 6 (WD group) for ANOVA Friedman’s test. Squares indicate the median values. Boxes indicate the IQR. Whiskers indicate the nonoutlier range. Circles indicate outliers, and asterisks indicate extreme values—the values beyond the nonoutlier range. pH, The activity of hydrogen ions; pCO_2_, Carbon dioxide partial pressure; pO_2_, Oxygen partial pressure; cHCO_3_^−^, Bicarbonate concentration; Be(ecf), Base excess in the extracellular fluid; SaO_2_, Oxygen saturation of hemoglobin; lactate, Lactate level.

The results of the *ABG* parameters examined for the Friedman ANOVA test are presented in Fig. 3. The comparison of all *ABG* samples in the *WS* and *WD* groups showed that statistically significant differences between groups were found in relation to *pH*, pO_2_, and SaO_2_ (Table 3). The presented results show the connection between those parameters according to the well-known acid–base equilibrium model, and there is confirmation of the reliability and validity of the research approach. The observations concerning steady changes in the time of *AGB* parameters in groups *WS* and *WD* showed that there was no statistically significant difference between the values of these parameters over time (independent variable) (Table 3). Despite the lack of these statistically significant differences, in the *WD* group, *Be(ecf)* showed a positive Spearman correlation coefficient (r = 0.433), which means that the *Be(ecf)* values increased over time during the experiments. In relation to the *WS* group, the Spearman correlation coefficients were also positive for cHCO_3_^−^ and *Be(ecf)* (0.509 and 0.561, respectively). In the *WS* group, the opposite trend was found for lactate levels (r = − 0.393), which means that lactate values decreased over time.

**Table 3:** Arterial blood gas analysis from animal experiments.

Variable	Unit	Mann–Whitney U Test			WS		*r*	WD		*r*
		WS (n = 31^#^)	WD (n = 29)		Friedman Test			Friedman test		
		Median [IQR]	Median [IQR]	*p*	*p*	*W*		*p*	*W*	
pH		7.39 [7.35 - 7.43]	7.42 [7.41 - 7.44]	.023*	.760	0.155	0.075	.554	0.156	0.215
pCO_2_	mmHg	54.8 [49.8 - 60.5]	52.7 [48.7 - 54.2]	.169	.371	0.355	0.164	.661	0.120	-0.126
pO_2_	mmHg	195.4 [184.6 - 203.9]	169.6 [147.3 - 192.9]	.007*	.139	0.578	-0.341	.451	0.184	-0.028
cHCO_3_ ÷	mmol/L	33.1 [31.6 - 35.0]	33.7 [32.8 - 35.1]	.395	.057	0.763	0.509	.142	0.344	0.324
Be(ecf)	mmol/L	8.3 [6.9 - 10.1]	9.4 [7.9 - 10.8]	.184	.082	0.689	0.561	.237	0.277	0.433
SaO_2_	%	99.7 [99.6 - 99.7]	99.4 [98.6 - 99.6]	< .001*	.318	0.393	-0.137	.294	0.247	-0.052
Lactate	mmol/L	0.70 [0.51 - 0.92]	0.71 [0.46 - 0.95]	.947	.339	0.378	-0.393	.634	0.128	-0.184

Summary for two animal groups ÷ pairs with similar weights (*WS*) and pairs with different weights (*WD*). Mann‒Whitney U test—between the groups for all blood samples. Friedman Test—analysis of blood samples across time by the ANOVA Friedman test for each group separately.

pH, The activity of hydrogen ions; pCO_2_, Carbon dioxide partial pressure; pO_2_, Oxygen partial pressure; cHCO_3_^÷^, Bicarbonate concentration; Be(ecf), Base excess in the extracellular fluid; SaO_2_, Oxygen saturation of hemoglobin; lactate, Lactate level; IQR, Interquartile range; *W*, Kendal’s coefficient of concordance; *r*, Spearman’s rank correlation coefficient.

*p < 0.05 considered statistically significant.

^#^n = 30 for Be(ecf).

### Laboratory experiments: effects of changing resistance and compliance in one artificial lung on flow and pressure in the second lung

We performed the tests of mechanical property changes (named later 'events') in a first artificial lung (*AL*) to the flow and pressure changes in a second *AL* (the scheme of the laboratory setup is presented in Fig. S1, and its picture is presented in Fig. S2). The results are presented in Table 4 and Figs. S3–S8.

**Table 4:** Step changes (events) of the resistance (*R*) and compliance (*C*) in object *#1* (left artificial lung).

	P1 (pre)	P2 (pre)	P1 (post)	P2 (post)	dP1	dP2	dP1%	dP2%	V1 (pre)	V2 (pre)	V1 (post)	V2 (post)	dV1	dV2	dV1%	dV2%
**RR = 12 (1/min)**																
R5 to R20	16.7	15.7	17.7	15.8	1.0	0.1	6.0	0.6	638	583	630	598	-8	15	-1.3	2.6
R5 to R50	16.5	16	30.4	16.1	13.9	0.1	84.2	0.6	614	603	653	597	39	-6	6.4	-1.0
R5 to R200	16.8	15.7	84.5	14	67.7	-1.7	403.0	-10.8	641	581	516	466	-125	-115	-19.5	-19.8
R5 to R0	16.8	15.7	1	15.3	-15.8	-0.4	-94.0	-2.5	639	584	627	590	-12	6	-1.9	1.0
C75 to C60	16.6	15.7	22.2	15.7	5.6	0	33.7	0.0	637	588	642	589	5	1	0.8	0.2
C75 to C25	16.7	15.6	27.5	15.5	10.8	-0.1	64.7	-0.6	646	581	658	563	12	-18	1.9	-3.1
C60 to C75	21.65	21	17	20	-4.7	-1	-21.5	-4.8	619	606	638	574	19	-32	3.1	-5.3
AL to Bag	16.45	15.8	36.55	15.8	20.1	0	122.2	0.0	631	571	657	607	26	36	4.1	6.3
**RR = 18 (1/min)**																
R5 to R20	13.8	13.5	15	13.6	1.2	0.1	8.7	0.7	430	389	428	399	-2	10	-0.5	2.6
R5 to R50	13.9	13.5	28.4	13.8	14.5	0.3	104.3	2.2	422	400	442	406	20	6	4.7	1.5
R5 to R200	14	13.5	85	13.6	71.0	0.1	507.1	0.7	423	401	457	406	34	5	8.0	1.2
R5 to R0	13.8	13.6	1	12.8	-12.8	-0.8	-92.8	-5.9	424	400	438	387	14	-13	3.3	-3.3
C75 to C60	14	13.5	18.5	13.5	4.5	0	32.1	0.0	412	400	426	393	14	-7	3.4	-1.8
C75 to C25	14	13.4	24.7	13.5	10.7	0.1	76.4	0.7	425	401	438	409	13	8	3.1	2.0
C60 to C75	18.5	18	14.6	17.1	-3.9	-0.9	-21.1	-5.0	431	400	436	379	5	-21	1.2	-5.3
AL to Bag	14	13.4	26.8	13.5	12.8	0.1	91.4	0.7	435	390	438	413	3	23	0.7	5.9
**RR = 24 (1/min)**																
R5 to R20	12.4	12.2	13.5	12.3	1.1	0.1	8.9	0.8	321	302	320	303	-1	1	-0.3	0.3
R5 to R50	12.4	12.3	27.7	12.5	15.3	0.2	123.4	1.6	321	305	341	312	20	7	6.2	2.3
R5 to R200	12.5	12.3	84	13.5	71.5	1.2	572.0	9.8	321	305	363	366	42	61	13.1	20.0
R5 to R0	12.4	12.3	1	11.3	-11.4	-1	-91.9	-8.1	321	306	334	292	13	-14	4.0	-4.6
C75 to C60	12.5	12.3	16	12.2	3.5	-0.1	28.0	-0.8	321	305	336	300	15	-5	4.7	-1.6
C75 to C25	12.5	12.3	21.5	12.4	9.0	0.1	72.0	0.8	319	300	331	310	12	10	3.8	3.3
C60 to C75	16.1	15.7	13	14.9	-3.1	-0.8	-19.3	-5.1	328	302	335	294	7	-8	2.1	-2.6
AL to Bag	12.5	12.3	21.6	12.3	9.1	0	72.8	0.0	318	302	327	310	9	8	2.8	2.6

All pressures refer to the respiratory peak pressure, expressed in cmH_2_O. All volumes refer to the respiratory tidal volume, expressed in ml.

pre, Index referring to the variable before the event; post, Index referring to the variable after the event; *P1*, *P2*, Pressures for object #1, #2, respectively; *V1*, *V2*, Volumes for objects #1, #2, respectively; *dP1* = *P1*(post)–*P1*(pre); *dP2* = *P2*(post)–*P1*(pre); *dP1*%, Relative percentage error for *dP1*; *dP2*%, Relative percentage error for *dP2*; *dV1* = *V1*(post)–*V1*(pre); *dV2* = *V2*(post)–*V2*(pre); *dV1*%, Relative percentage error for *dV1*; *dV2*%, Relative percentage error for *dV2*; “*RX to RY*” refers to the initial resistance value (*X*) and the resistance value after the event (*Y*) (for example: if X = 5 and Y = 200, then its “R5 to R200”), when the compliances are default (*C* = 75) and constant; “CA to CB” refers to the initial compliance value (*A*) and the compliance after the event value (*B*) (for example: if A = 75 and B = 25 then its ‘C75 to C25’), when the resistances are default (*R* = 5) and constant; “AL to Bag” refers to the exchange of the artificial lung (*R* = 5, *C* = 75) with the 2 L respiratory bag (*R* ~ 0, *C* ~ 15); *R* values are expressed in mbar/L/s; *C* values are expressed in ml/mbar; the Ventil ratio is approximately 1:1.

The pressure change (*dP*), defined as the difference between the *PIP* in one of the Ventil system’s output channels before and after the event, was obviously changed in the affected *AL* (*dP2*). The percentage relative error for *dP2* (*dP2%*) was proportional to the simulated *R* and *C* changes. For the tested (not affected) *AL*, this pressure change (*dP1*), expressed as a percentage relative error (*dP1%*), was less than 10% for all cases, except *'R5 to R200'* for the *RR* = 12 event, where it was 11%. The *TV* change (*dV*) for affected and tested *AL* (*dV1* and *dV2,* respectively) expressed as the percentage relative errors (*dV1%* and *dV2%,* respectively) were also less than 10% with two exceptions. One exception was for *'R5 to R200'* for *RR* = 12. In this case, the inspiration was finished early by the ventilator due to the very high pressure in the ventilator output port (Fig. S3). Therefore, *dV1*% and *dV2*% were at the level of − 20%. The second exception was for ‘R5 to R200’ for *RR* = 24. In this case, the tidal volumes were 13% and 20% higher (*dV1*%, *dV2*%) for the tested and affected *AL,* respectively. Because the Ventil apparatus only splits the gas flow, the *TV* increase for both *ALs* in the last case could have been caused only by the work of the ventilator under this high-pressure condition in the breathing circuit.

These experiments show the strong Ventil endurance in maintaining division of the minute ventilation when the respiratory system *R* and *C* parameters are changed (i.e., respiratory deterioration or improvement in a patient). Ventil stabilized the ventilation within a few respiratory cycles after an adverse event had occurred.

Other laboratory experiment results using the same setup (Figs. S1, S2) are described in the supplementary materials and are presented in Figs. S9–S15.

### Simulation of cross-contamination: laboratory tests

Research on the transmission of particles in the dual tract respiratory system with Ventil was carried out in two stages with the use of (1) aqueous solutions of fluorescent compounds and (2) suspension of the *SARS-CoV-2* coronavirus phantom in the form of fluorescent nanospheres. The presence of test substances and nanoparticles was examined at test points *T1*, *T2* and *T3*. The results are presented in Supplement R1 in the form of a technical report on the transmission of solutions/nanoparticles in two respiratory branches of the Ventil system. There were no fluorescein traces on the postfiltering side of the filters located at test points *T1*, *T2* and *T3*. No traces of fluorescein were observed on the postfiltering surface filter surface for the filter placed at test point *T3*. Furthermore, in our experiments under most critical conditions, when electrostatic filter *F* and the *PEEP* valve were removed, viral phantoms in the form of fluorescently labeled nanospheres with a diameter of 100 nm in the stream of air exhaled from the left lung did not spread further than the filtering surface of the filter at test point *T3*.

In summary, based on the conducted technical studies related to the assessment of the possibility of transmission of solutions/suspensions in two respiratory branches of the Ventil system, it was found that under the conditions of the experiment with the use of fluorescent solutions and suspensions, no transmission occurred.

## Discussion

The results in the animal model show that the ventilation of two animals can be effectively continued for at least 24 h by the ventilator and the Ventil system, independent of the body weight difference in the pair. The oxygenation, ventilatory status and acid–base balance of the animals were stable during the experiments. The animals’ weight difference was effectively compensated using Ventil use by adjusting the *TV* according to their need. Subject-specific minute ventilation was maintained by changing the ventilator’s MV and the Ventil device’s division ratio. The EtCO_2_ levels were elevated due to the lung-protective strategy applied in the experiments.

Evaluating *ABG* parameters, we focused on the direction and range of changes over time. SaO_2_, *pH* and lactate concentrations were maintained in the normal range. The results of pO_2_ exceeded the normal level of 100 mmHg in both groups. Our results in the *WS* group are comparable with the results reported by Bitelia et al. for the group of pigs ventilated with oxygen and compressed air FiO_2_ 0.5/air^32^. The *WD* group showed lower values than the *WS group* but within the normal range for all time points. The pCO_2_ showed high values that are comparable with the values reported by others^32,33^. The value of cHCO_3_^−^ remained at a high level, with a trend of changes similar to pCO_2_. The high values of cHCO_3_^−^ and the same trend as pCO_2_ may indicate that elevated cHCO_3_^−^ is a metabolic response to pCO_2_, especially when changes in blood oxygenation and a low lactate concentration indicate adequate tissue oxygenation. Taking into account the time of observation, we presume that two of three compensatory systems, extracellular and respiratory, can be responsible for this finding. The renal compensatory system acts slowly to compensate for acid–base balance^34^. Among several factors that can be responsible for the obtained results of pCO_2_ and cHCO_3_^−^*,* the primary cause should be sought in disorders of CO_2_ exchange due to changes in the position of the pigs from the side to the back, which can induce disturbances in gas exchange between perfusion and ventilation.

The *CT* scan analysis carried out before and after long-term ventilation did not reveal serious injuries, such as emphysema, pneumothorax and pleural effusion caused by the ventilation procedure, with the exception of the animal with emphysema in which left-sided pneumothorax and both-sided pneumothorax were indicated before and after the experiment, respectively. This animal had *ΔP* equal to 12, while other animals had *ΔP* equal to or less than 10. This result suggests that the safe *ΔP* indicator for ventilated pigs is < 10 cmH_2_O.

In animal experiment 3, the progressive changes in the lung compliance of subject *#1* due to saline instillation did not affect the ventilation of unaffected subject *#2*; however, these changes were not strong because 200 ml of saline was not a large volume in comparison to the affected subject’s weight. In Experiments 3 and 6, replacing an animal with a 2 L respiratory bag (no resistance, very small compliance) increased the PIP up to 17% in unaffected subjects and changed the TV as much as 25% in ventilated subjects. Because the MV of the ventilator was constant, these changes are related to Ventil’s division ratio. In laboratory experiments for ‘AL to Bag’ events, PIP changes in the unaffected AL were less than 1%, and TV changes for both ALs ranged from 0.7 to 6.3%. However, in the animal experiments, the inspiratory flow of the ventilator was 80 L/min, while it was 40 L/min in the laboratory experiments. This means that Ventil is more accurate and more load independent for lower inspiratory flows. Less performance for higher flows can be related to the higher dispersion of two analog flow sensors in the Ventil. If these sensors’ pressure-flow characteristics are load impedance dependent, then flow level inequality is produced, and the volume division is not exactly as expected. However, the laboratory results in maintaining *MV* in both channels (when *R* and *C* are changed) are still acceptable and can be somewhat corrected by Ventil’s flow division knob.

### Limitations of the shared ventilation by Ventil

As in other solutions, in the case of ventilation by Ventil, subjects share the respiratory rate, inspiratory time, pause time and oxygen content set in the ventilator, which limits the respiratory therapy capabilities. Moreover, the respiratory activities of both ventilated subjects may lead to dynamic changes in the pressure of the airways and significant disturbances in the ventilation mechanics of one or both subjects. Thus, subjects with maintained respiratory function need deep sedation^10,11,16^.

Additional mechanical *PEEP* valves can be used with Ventil to differentiate intrinsic *PEEP* (*PEEPi*) levels during therapy. In animal experiments, mechanical, fixed *PEEP* valves were used. However, neither ventilator that we used cooperated when fixed *PEEP* valves of 5 cmH_2_O or higher were used. The occlusion alarm was indicated by both ventilators immediately. The 2.5 and 0 cmH_2_O *PEEP* valves (only one-way valves) worked correctly. Therefore, extrinsic *PEEP* (*PEEPe*) values were also set in the ventilator. *PEEPi* in the lungs of the subject at the end of the expiration is a sum of set *PEEPe* and mechanical *PEEP* valves. Other authors in their studies neglected differential *PEEP* levels^12^ and used only a shared *PEEP* set in a ventilator. However, various *PEEP* levels for ventilated patients in shared ventilation systems are an open issue.

### Safety indications

The shared ventilation in the context of the treatment of *COVID-19* patients provoked a global discussion on the limitations and safety of such approaches in emergencies^38,39^. Most of the studies of shared ventilation were based on laboratory experiments in which only test lungs were used^4,7,12,16,36^. Only a few animal experiments^5,8–10^ or preliminary tests on a few pairs of patients^35,37^ were carried out. Further clinical studies focused on safety and effectiveness are necessary. Below, we discuss some safety indications.

Ventilator alarm and monitor management in the case of shared ventilation is a challenge^10,16,35^. External monitoring systems are necessary for ventilated subjects, as has also been confirmed by other authors^10–12^. For 1:1 division, the peak pressure difference between the Ventil outputs and that measured by the ventilator can be as high as 20 cmH_2_O for a flow level of approximately 100 L/min (Fig. S15). Peak pressure values in the ventilator can then be elevated, and the high-pressure alarm must be corrected. The Ventil displays only the mean pressure in the output ports and the mean flow for both channels. A low mechanical ventilation alarm is also essential to prevent leakage in the whole respiratory circuit^35^.

In comparison to other shared ventilation solutions, Ventil is an active system. However, its malfunction should not introduce rapid mechanical ventilation changes for ventilated subjects (Figs. S9–S14). A dividing head in the Ventil is constructed to make it mechanically impossible to close one of the channels or both of them, even in the case of electronic and control system failure. Moreover, we did not use pressure relief valves in our animal experiments due to continuous supervision and external subject monitoring. However, they must be applied in the breathing circuit in clinical settings^10^. These valves prevent excessive ventilation in patients. Using Ventil, the operator must also carefully manage when an extensible, flexible tube between the ventilator and the Ventil is used. In our laboratory experiments, too-rapid stretching of this tube caused a ventilator (Bennett 840) malfunction. The ventilator’s safety valve was activated, and the ventilator was switched into the service mode. This was probably due to the negative pressure created between the ventilator and the Ventil apparatus.

The complexity of the setup (Fig. 1) can also be problematic for medical staff. An additional device with a set of additional tubes and connectors increases the risk of leakage or a wrong connection. Some components in the setup can be reduced by modifying the Ventil’s design, but the setup in shared ventilation will always be more complex than “one patient – one ventilator”.

### Cross-contamination in shared ventilation by Ventil

Similar to the results obtained by other authors, there was no transmission of fluorescent dye solutions—trypan blue^10^, fluorescein or methylene blue—as indicated in our study presented in Report R1 in the supplementary materials. Concerning the studies described in^10^, which include continuous nebulization of 5 ml of trypan blue solution for 10 min and visual observation of the filters, in our experiment, in addition to the use of fluorescein and methylene blue solutions, we proposed a pathogen transmission study method using phantoms of the virus in the form of nanospheres with a diameter of 100 nm internally labeled with fluorescent dyes, the concentration of which in the administered suspension was approximately 24.1012 particles/3 mL, which corresponds to a viral titer of ca. 1000-fold excess with respect to *SARS-CoV-2* titer after a 5-day incubation of infected *Calu3* cells[^21^]. The proposed method using nanoparticles can be safely carried out in the laboratory environment, obtaining more specific (in relation to the acquisition from experiments with solutions) preliminary information about the possibility of cross-transmission in the respiratory tract during independent ventilation of artificial lungs.

## Conclusions

In conclusion, Ventil might be considered to ventilate two patients by one ventilator with an acceptable safety level. It meets the same challenges as other shared ventilation systems. However, Ventil automatically stabilizes the patients' minute ventilation and is highly invulnerable to their respiratory resistance and compliance changes. Notwithstanding, the Ventil system is feasible for differential lung ventilation and can still be applied for respiratory therapy in cases involving asymmetrical lung pathology or thoracic surgery. Additionally, it can be used in emergency situations when the lack of ventilatory support for a large number of patients is expected, such as during terrorist attacks or widespread diseases affecting respiratory function.

## Supplementary Information

Supplementary Information.

## Data Availability

All data, except CT scans, are available in the main text or the supplementary materials. The CT scans are available from the corresponding author upon reasonable request.
