# Supplementary material for: Automatic air volume control system for ventilation of two patients using a single ventilator: a large animal model study
Source: Sci Rep. 2022 Dec 30;12:22591. doi: 10.1038/s41598-022-26922-4 (PMC9801355; doi:10.1038/s41598-022-26922-4)
Supplement: Supplementary file 1 — Supplementary Information. [file 41598_2022_26922_MOESM1_ESM.pdf]

Automatic air volume control system for ventilation of two patients using a single ventilator: a large animal model study

Krzysztof Zieliński[1*], Barbara Lisowska[2], Katarzyna Siewruk[3], Maria Sady[3,7], Karolina Ferenc[3,7], Maciej Barwijuk[4], Jarosław Olszewski[3,7], Krzysztof Anusz[3], Artur Jabłoński[3,7], Magdalena Gajewska[3,5], Piotr Okrzeja[1], Marcin Michnikowski[1], Dorota G. Pijanowska[1], Krzysztof Pluta[1], Elżbieta Remiszewska[1], Marek Darowski[1], Romuald Zabielski[3,7], Adam Liebert[1], Katarzyna Kramek-Romanowska[6], Anna Stecka[1], Maciej Kozarski[1], Raman Pasledni[1], Zdzisław Gajewski[3,7†], Piotr Ładyżyński[1†]

[1]Nalecz Institute of Biocybernetics and Biomedical Engineering, Polish Academy of Sciences, Warsaw, Poland

[2]Department of Anesthesiology and Intensive Medical Care, National Geriatrics, Rheumatology and Rehabilitation Institute, Warsaw, Poland

[3]Warsaw University of Life Sciences – SGGW, Veterinary Research Center, Center for Biomedical Research and Research Center for Regenerative Medicine, Warsaw, Poland

[4]I Department of Anaesthesiology and Intensive Care, Medical University of Warsaw, Warsaw, Poland

[5]Medical University of Warsaw, Warsaw, Poland

[6] Faculty of Chemical and Process Engineering Warsaw University of Technology, Warsaw, Poland

[7] Warsaw University of Life Sciences – SGGW, Center of Translational Medicine, Warsaw, Poland

† These authors contributed equally to this work as senior authors of the paper

SUPPLEMENTARY RESULTS

Laboratory simulations of the Ventil device malfunction:

We performed tests to simulate a Ventil device malfunction when the function of automatic stabilization and division of the minute ventilation is abandoned. It covers the following situations: the electronic power failure, including a medical power supply unit failure, the temporary power grid failure in a medical facility, as well as the Ventil control electronic system failure. In a stable situation, we unplugged the power cord from the Ventil socket what immediately stopped the Ventil device. We made this "unplug" experiment in different respiratory stages, i.e., during the inspiration, end-inspiratory pause, and expiration. We repeated these experiments, but for the situation that powered off the Ventil device is powered on by plugging the power cord to the socket, simulating, for example, power grid restoration in the hospital. The results are presented in figures S9-S14. Additionally, we have successfully tested the Ventil device, for its continuous working, for two weeks.

The above results show that in a steady state when the Ventil stabilized division of the minute ventilation, the potential malfunction of the system could be safe for patients. The Ventil still divides the flow, and turning the power off and then on has virtually no effect on the flow and pressure signals. The Ventil dividing head is in a fixed position when the device is not powered; thus, it can still divide the flow but passively and potentially less accurate. However, in the case of the patient's respiratory improvement or deterioration, the position of the dividing head will not be changed until the power supply is restored. Therefore, in this case, the Ventil operates as a passive splitter.

Static pressure-flow characteristics

We determined the static inspiratory peak pressure drop (*dPpeak*) – maximal flow characteristics for the Ventil device. For 1:1 flow division, the *dPeak* between the Ventil input and two outputs (*dPeak1*, *dPeak2*) were collected. The experiments were done for various minute ventilation values, respiratory rate values, and inspiratory to expiratory ratios as well. The characteristics are presented in figure S15. *Flow Max* in this figure is the maximal flow set in ventilator (volume-controlled mode with rectangular inspiratory flow shape), while the *dPeak1* is the difference between the inspiratory peak pressure obtained on the ventilator and measured in the Ventil left output channel and the *dPeak2* is the difference between the same peak pressure from the ventilator and measured in the Ventil right output channel. We have obtained very linear characteristics within the measured range with a coefficient of determination *R2* close to 1. We must point out that for higher flow rates, the differences between the pressure in the individual breathing circuit and the pressure measured by the ventilator can be up to 20 cmH2O for 1:1 ventilation. The peak pressures in the ventilator can be higher than typical and trigger too high-pressure alarms. Pressure alarms specific management is required then due to elevated peak pressures in the ventilator.

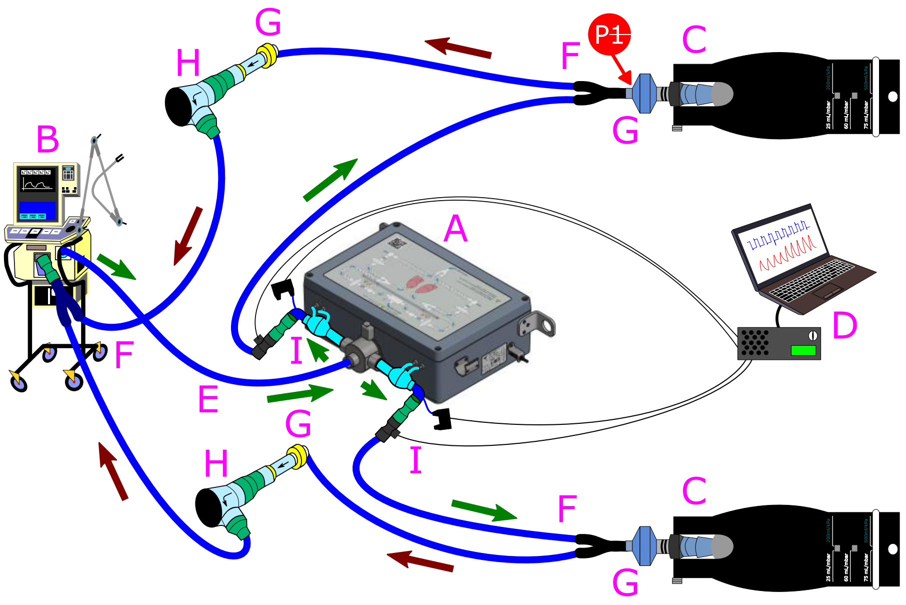

Fig. S1.

Laboratory experiments test bench. A, Ventil device; B, ventilator; C, artificial lung; D, data acquisition and measurement system; E, adult polypropylene extendible limb 22F-22F; F, adult polypropylene anesthesia extendible breathing circuits with Y-pieces; G, antibacterial and antiviral filter; H, positive end-expiratory valve with one-way valve and set of connectors; I, pressure and flow sensors; *P1*, disconnection point; green arrows – the flow direction during the inspiration; dark red arrows – the flow direction during the expiration

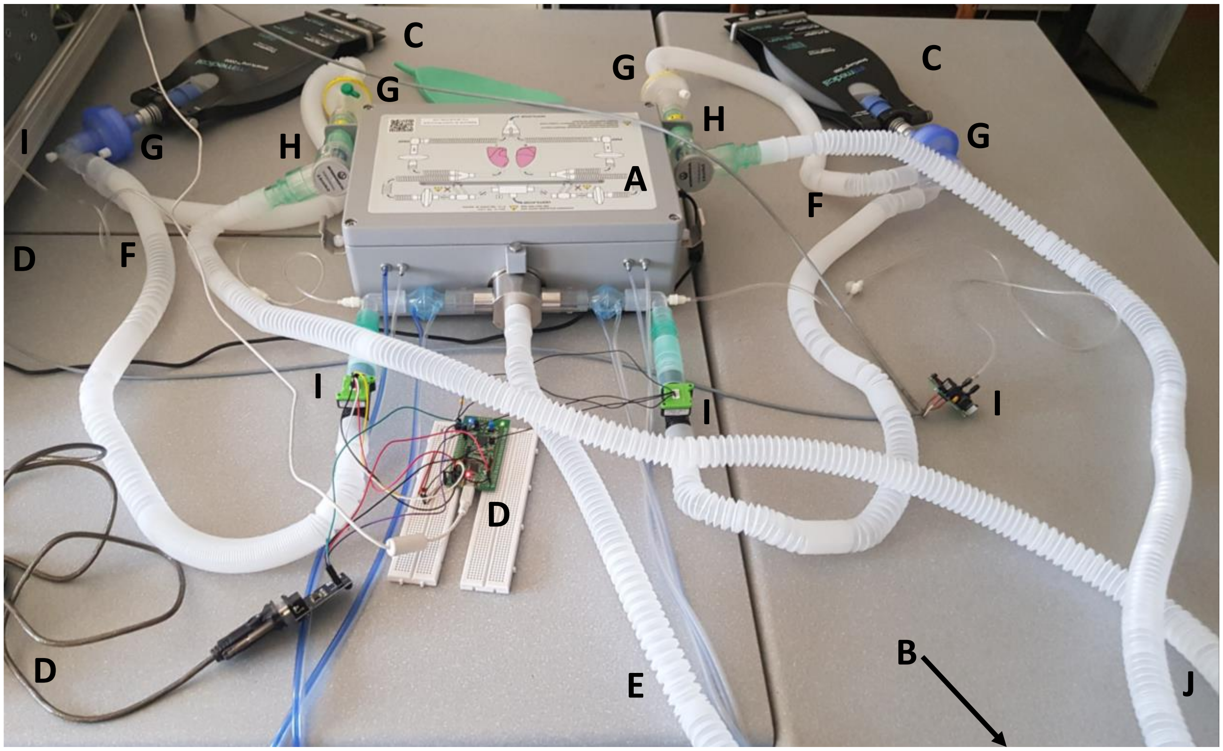

Fig. S2.

A picture of the laboratory test bench. A, Ventil device; B, ventilator (out of the picture); C, artificial lung; D, data acquisition and measurement system; E, adult polypropylene extendible limb 22F-22F (from the ventilator); F, adult polypropylene anesthesia extendible breathing circuits with Y-pieces; G, antibacterial and antiviral filter; H, positive end-expiratory valve with one-way valve and set of connectors; I, pressure and flow sensors; J, adult polypropylene anesthesia extendible breathing circuit with Y-pieces (to the ventilator)

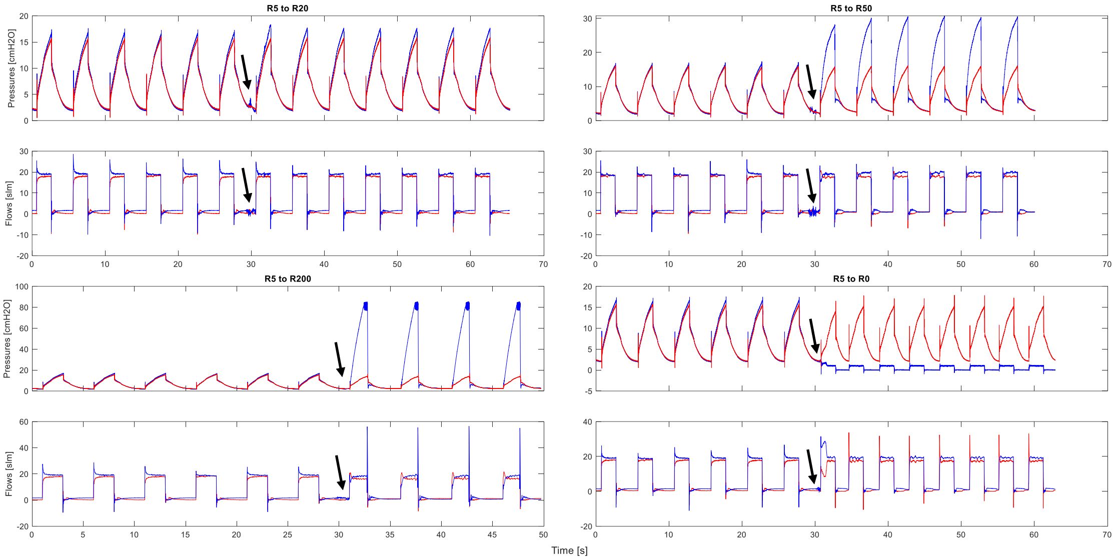

Fig. S3.

Step changes (events) of the resistance (*R*) in the *#1* object (artificial lung) on objects ventilation. Blue traces denote *#1* ventilated object, while red traces denote *#2* ventilated object. "*RX to RY*" refers to the initial resistance (*X*) and the resistance after the event (*Y*), when the compliances are default (*C*=75) and constant; *R* values are expressed in mbar/L/s; Ventil device ratio is about 1:1; Black arrow – a moment of the event. Respiratory rate *RR* = 12 1/min

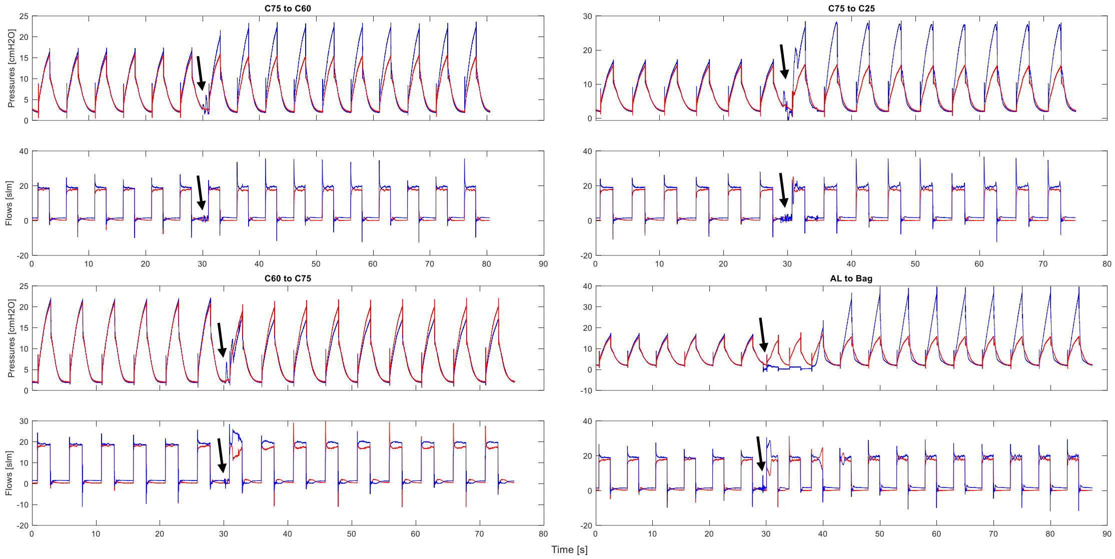

Fig. S4.

Step changes (events) of the resistance (*C*) in the *#1* object (artificial lung) on objects ventilation. Blue traces denote *#1* ventilated object, while red traces denote *#2* ventilated object. "*CA to CB*" refers to the initial compliance (*A*) and the compliance after the event (*B*), when the resistances are default (*R*=5) and constant; "*AL to Bag*" refers to the exchanging the artificial lung (*R*=5, *C*=75) to the 2L respiratory bag (R~0, *C*~15); *R* values are expressed in mbar/L/s; *C* are expressed in ml/mbar; Ventil device ratio is about 1:1; Black arrow – a moment of the event. Respiratory rate *RR* = 12 1/min

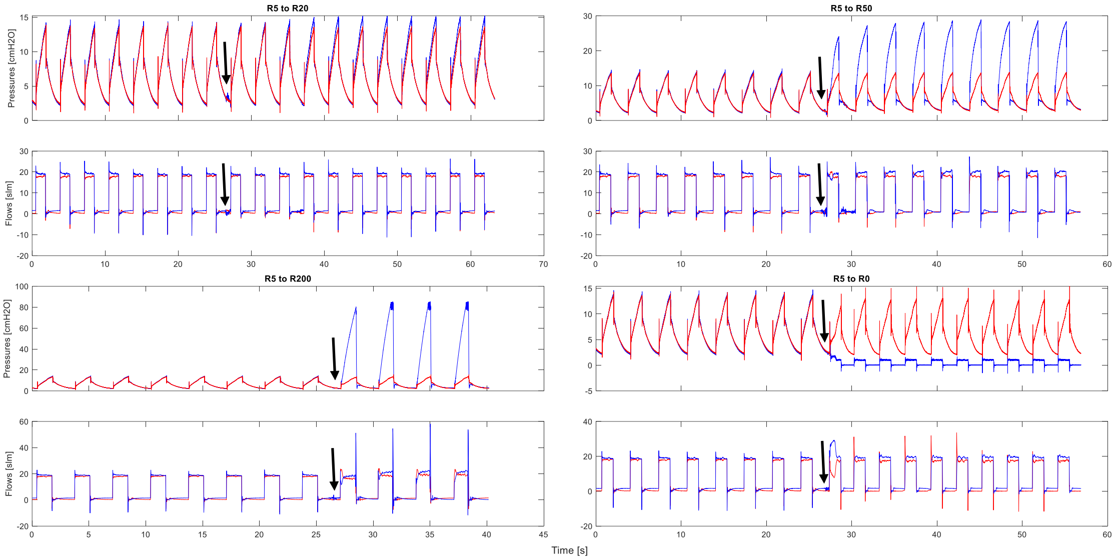

Fig. S5.

Step changes (events) of the resistance (*R*) in the *#1* object (artificial lung) on objects ventilation. Blue traces denote #1 ventilated object, while red traces denote *#2* ventilated object. "*RX to RY*" refers to the initial resistance (*X*) and the resistance after the event (*Y*), when the compliances are default (*C*=75) and constant; *R* values are expressed in mbar/L/s; Ventil device ratio about 1:1; Black arrow – a moment of the event. Respiratory rate *RR* = 18 1/min

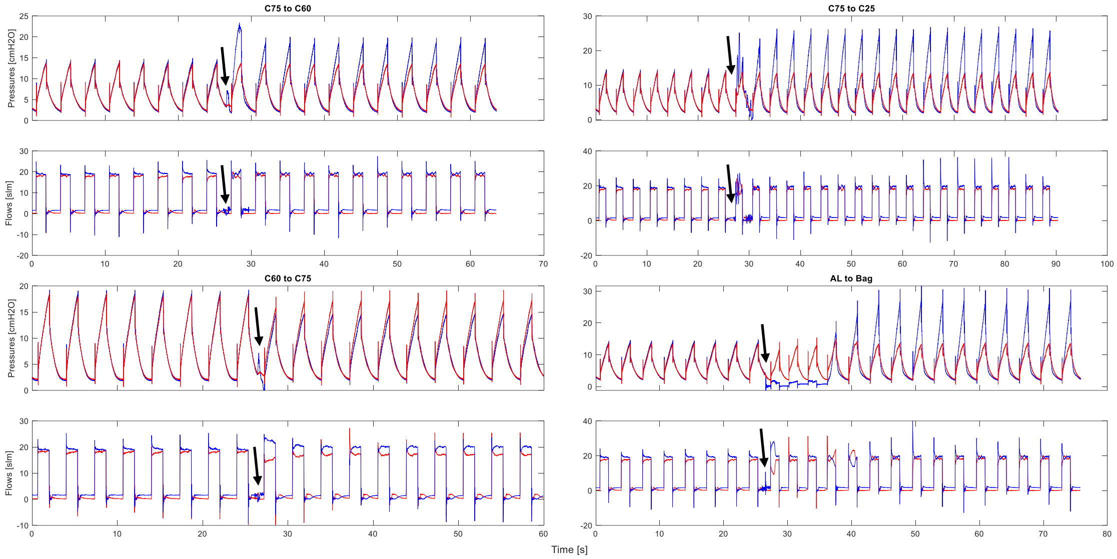

Fig. S6.

Step changes (events) of the resistance (*C*) in the *#1* object (artificial lung) on objects ventilation. Blue traces denote *#1* ventilated object, while red traces denote *#2* ventilated object. "*CA to CB*" refers to the initial compliance (*A*) and the compliance after the event (*B*), when the resistances are default (*R*=5) and constant; "*AL to Bag*" refers to the exchanging the artificial lung (*R*=5, *C*=75) to the 2L respiratory bag (*R*~0, *C*~15); *R* values are expressed in mbar/L/s; *C* are expressed in ml/mbar; Ventil device ratio is about 1:1; Black arrow – a moment of the event. Respiratory rate *RR* = 18 1/min

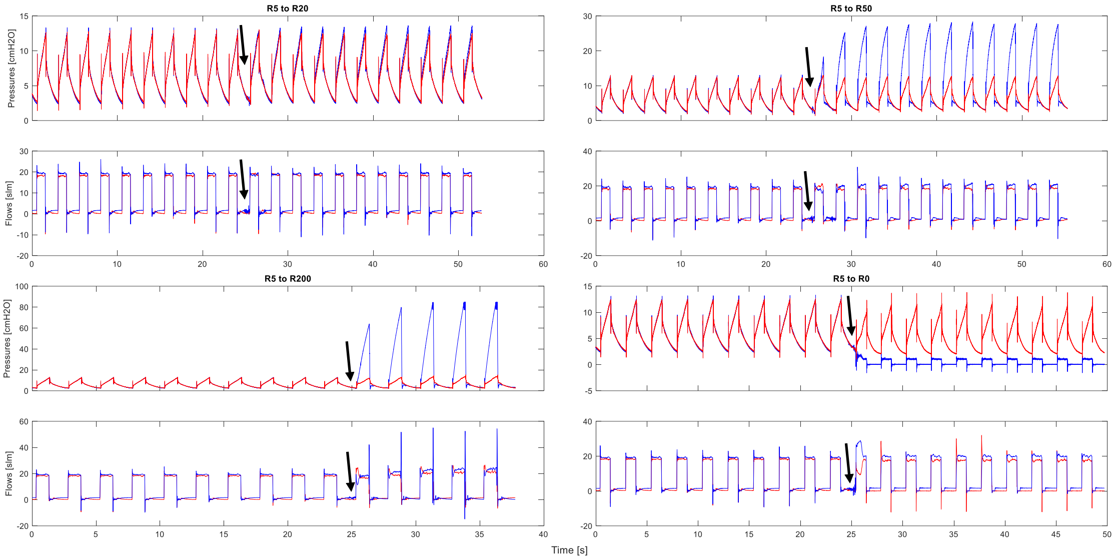

Fig. S7.

Step changes (events) of the resistance (*R*) in the *#1* object (artificial lung) on objects ventilation. Blue traces denote *#1* ventilated object, while red traces denote *#2* ventilated object. "*RX to RY*" refers to the initial resistance (*X*) and the resistance after the event (*Y*), when the compliances are default (*C*=75) and constant; *R* values are expressed in mbar/L/s; Ventil device ratio is about 1:1; Black arrow – a moment of the event. Respiratory rate *RR* = 24 1/min

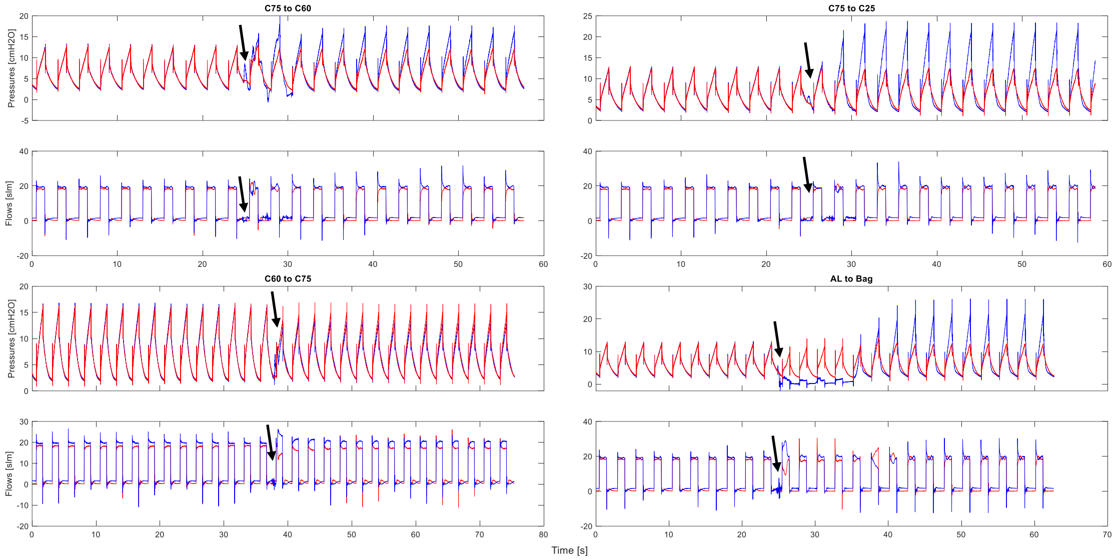

Fig. S8.

Step changes (events) of the resistance (*C*) in the *#1* object (artificial lung) on objects ventilation. Blue traces denote *#1* ventilated object, while red traces denote *#2* ventilated object. "*CA to CB*" refers to the initial compliance (*A*) and the compliance after the event (*B*), when the resistances are default (*R*=5) and constant; "*AL to Bag*" refers to the exchanging the artificial lung (*R*=5, *C*=75) to the 2L respiratory bag (*R*~0, *C*~15); *R* values are expressed in mbar/L/s; *C* are expressed in ml/mbar; Ventil device ratio is about 1:1; Black arrow – a moment of the event. Respiratory rate *RR* = 24 1/min

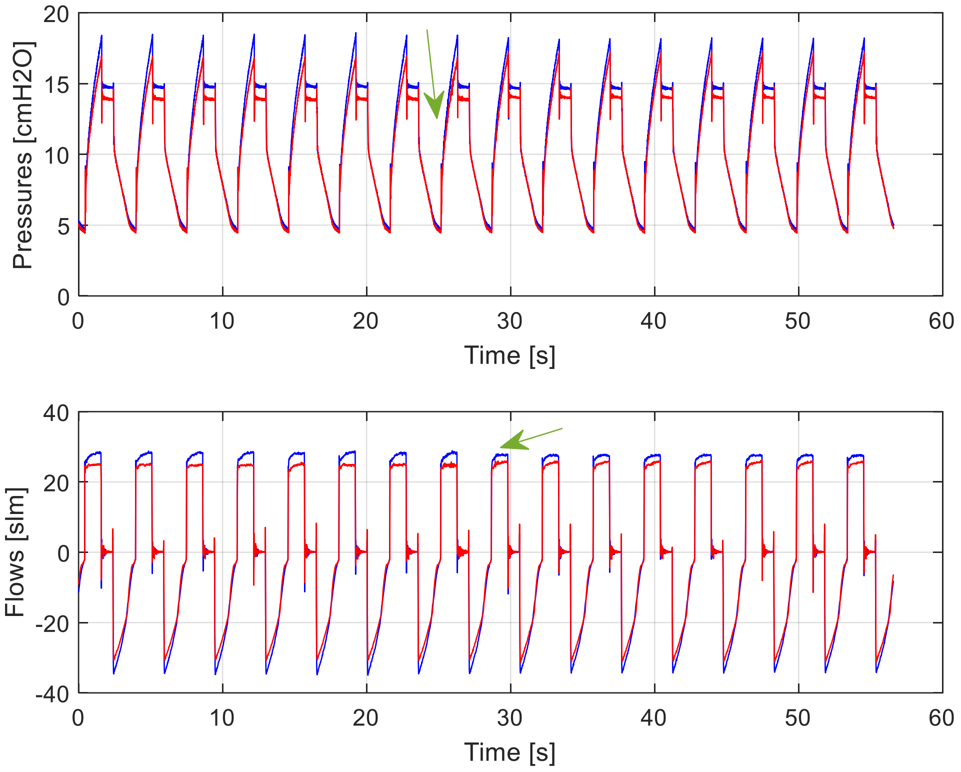

Fig. S9.

Simulation of the Ventil device malfunction. Pressures and flows in the Ventil outlets. Blue traces denote *#1* ventilated object (left outlet), while red traces denote *#2* ventilated object (right outlet); Green arrow – a moment of the Ventil device switched off during the inspiratory phase

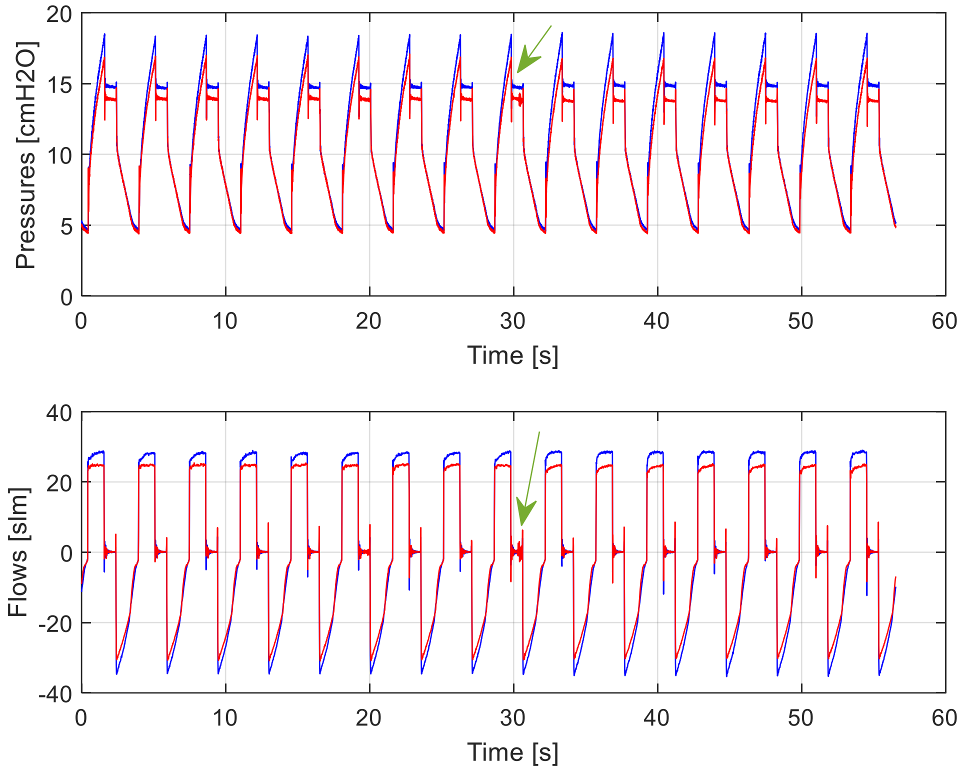

Fig. S10.

Simulation of the Ventil device malfunction. Pressures and flows in the Ventil outlets. Blue traces denote *#1* ventilated object (left outlet), while red traces denote *#2* ventilated object (right track); Green arrow – a moment of the Ventil device switched off during the end-inspiratory pause phase.

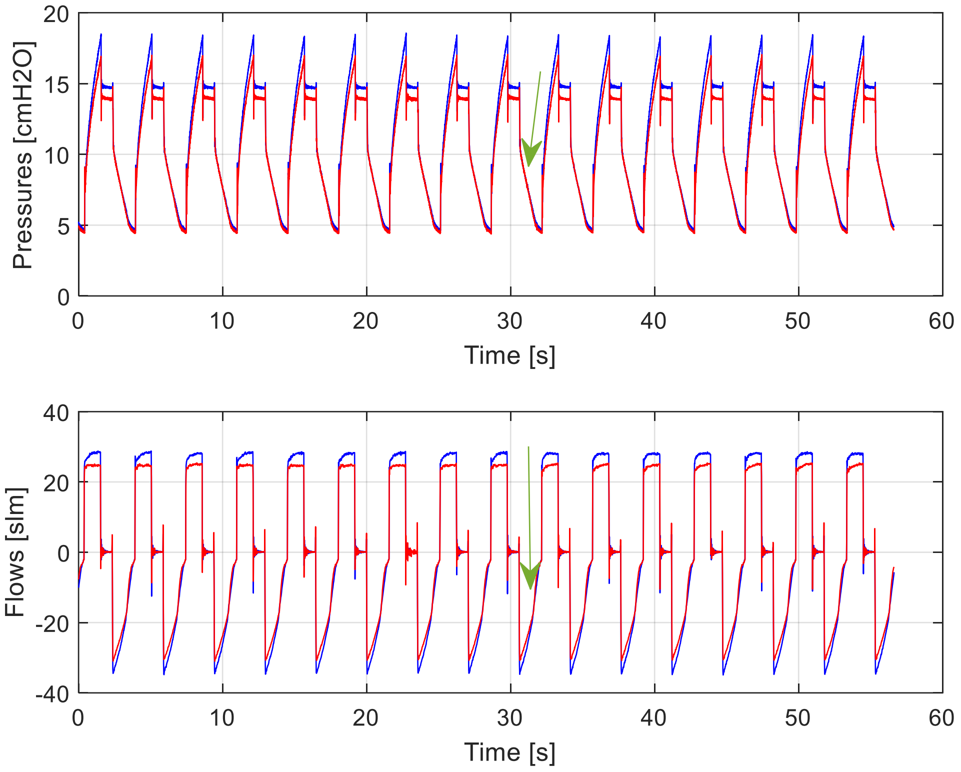

Fig. S11.

Simulation of the Ventil device malfunction. Pressures and flows in the Ventil outlets. Blue traces denote *#1* ventilated object (left outlet), while red traces denote *#2* ventilated object (right track); Green arrow – a moment of the Ventil device switched off during the expiratory phase.

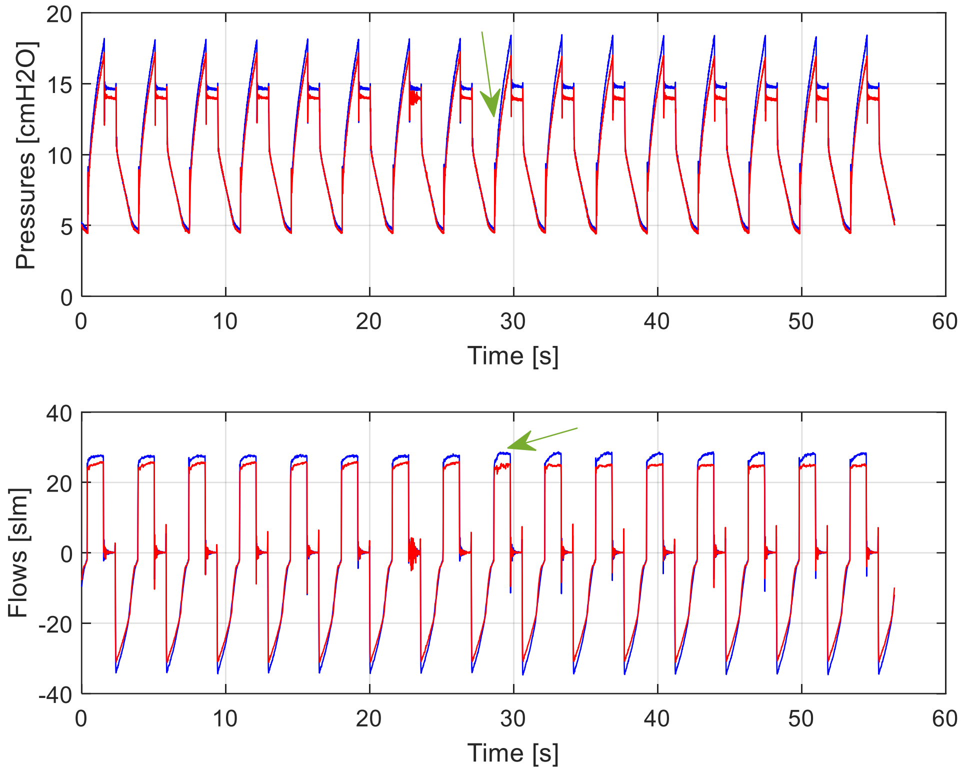

Fig. S12.

Simulation of the Ventil device malfunction. Pressures and flows in the Ventil outlets. Blue traces denote *#1* ventilated object (left outlet), while red traces denote *#2* ventilated object (right track); Green arrow – a moment of the Ventil device switched on during the inspiratory phase.

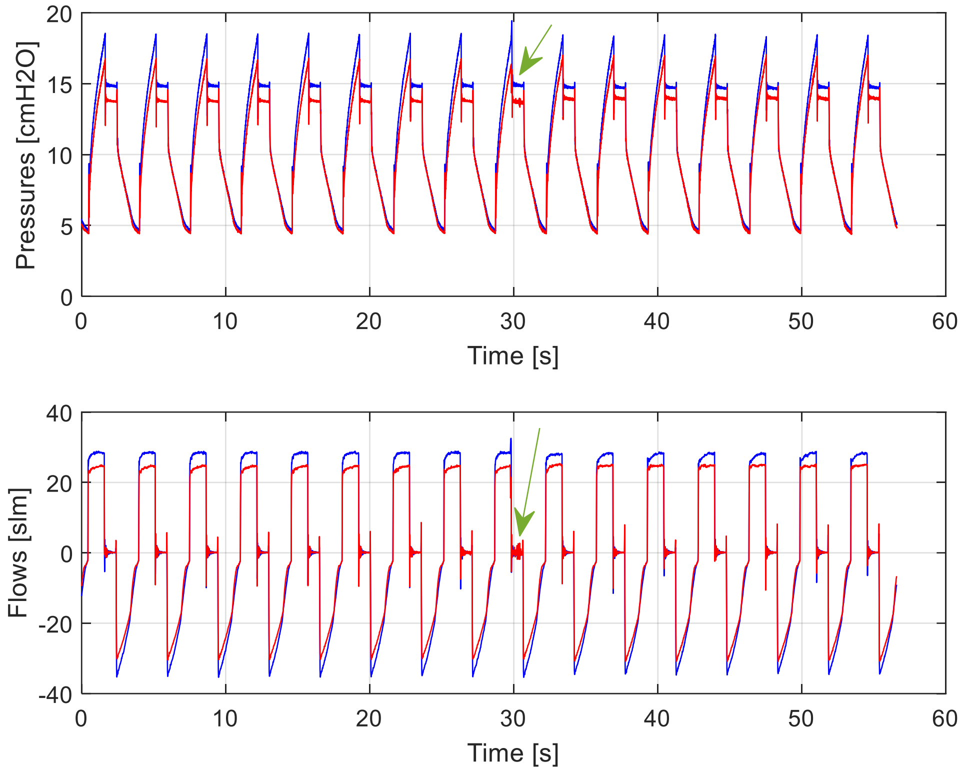

Fig. S13.

Simulation of the Ventil device malfunction. Pressures and flows in the Ventil outlets. Blue traces denote *#1* ventilated object (left outlet), while red traces denote *#2* ventilated object (right track); Green arrow – a moment of the Ventil device switched on during the end-inspiratory pause phase.

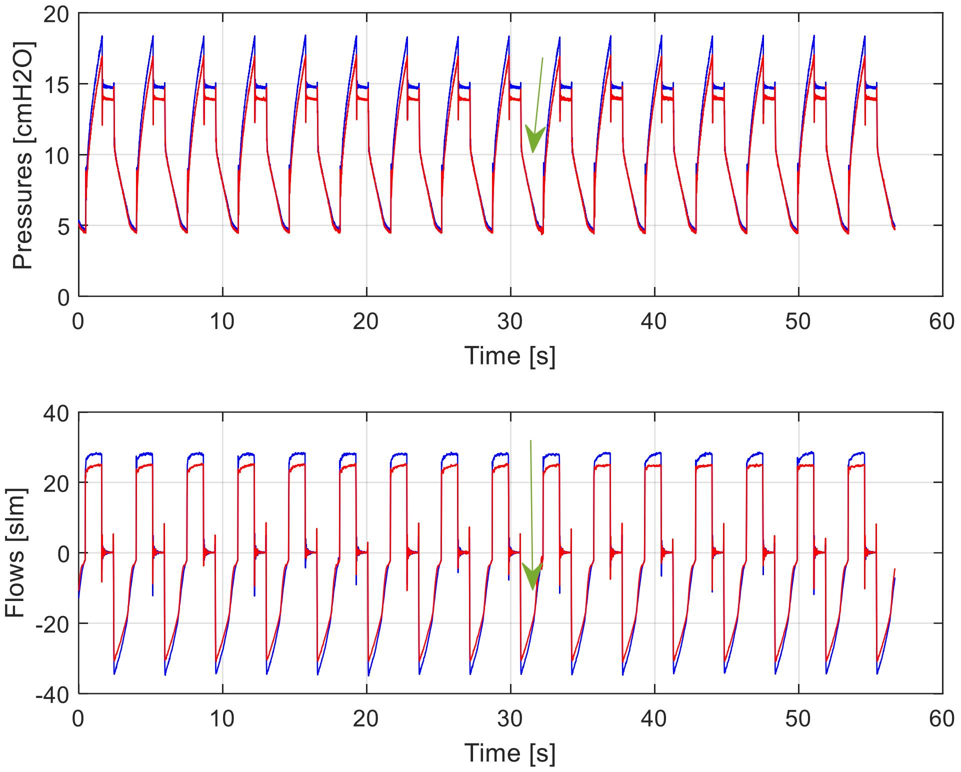

Fig. S14.

Simulation of the Ventil device malfunction. Pressures and flows in the Ventil outlets. Blue traces denote *#1* ventilated object (left outlet), while red traces denote *#2* ventilated object (right track); Green arrow – a moment of the Ventil device switched on during the expiratory phase.

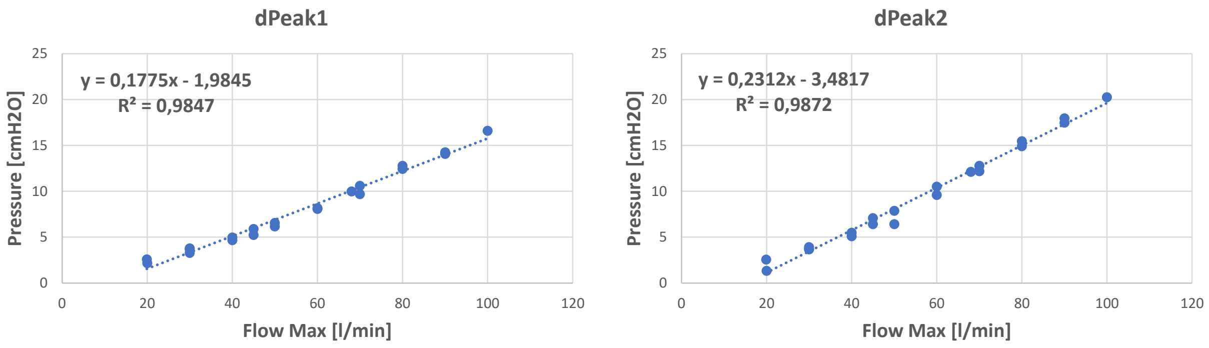

Fig. S15.

Static characteristics of the Ventil device. Pressure differences (*dPeak*) – flow (*Flow Max*) characteristics for the left (1) and the right (2) Ventil tracks. *dPeak* is defined as the difference between an inspiratory peak pressure measured in the ventilator and a peak pressure measured in the Ventil outlet (left or right); *Flow Max* is an inspiratory flow set in the ventilator. The Ventil flow division ratio is 1:1.

Nalecz Institute of Biocybernetics and Biomedical Engineering, Polish Academy of Sciences
Ks. Trojdena 4, 02-019 Warsaw, POLAND

Prof. Dorota Pijanowska, PhD, DSc
Krzysztof Pluta, PhD
Elżbieta Remiszewska
dpijanowska@ibib.waw.pl

Status: Technical report prepared for the needs of IBBE PAS.

Technical report (IBBE PAS, 22-07-2020)

– transmission of solutions/nanospheres in two respiratory branches of the Ventil system

The aim of the research was to test the possibility of nanoparticle transmission between the two respiratory branches of the Ventil system. The research was carried out in three stages, respectively, with the use of (1) fluorescent nanopowders, (2) solutions of fluorescent compounds, and (3) suspension of the SARS-CoV-2 coronavirus phantom in the form of fluorescent nanospheres.

For some viruses, such as HIV, the term viral load is a numerical expression of the quantity of virus in a given volume of fluid in a sample volume, e.g. blood, plasma, etc. In contrast, for viruses that are transmitted by airborne droplets, a better term is called viral shedding. Moving on to studies related to infections caused by the droplet method, which are not many, one article quantified the presence of influenza A and B viruses in the exhaled air. The rate of increase in the number of influenza virus RNA particles on the mask filter has been found to be 3.2 to 20 particles (virions) per minute (can be directly related to the number of viral particles) [*Fabian et al., 2008*]. However, the data described in the above articles do not allow for the determination of the minimum infectious dose of virus that can be transmitted by inhalation. In the absence of data on SARS-CoV-2 infection, a model based on the influenza virus infection model was adopted. The influenza virus and SARS-CoV-2 are single-stranded RNA viruses of similar size - approx. 120 nm.

In studies using the viral phantom in the form of fluorescently labeled nanospheres, the estimated phantom titer was determined from an exponential pattern of viral reproduction in the respiratory system.

Moreover, according to the data presented in the publication [*Chu et al., 2020*], it was indicated that in the culture medium of Caco2 and Calu3 cells infected with SARS-CoV-2 virus, 120 h after infection, the number of virus genome copies was determined to be $9.5 \cdot 10^{9}$ in 1 mL of supernatant. The multiplicity of infection (MOI), defined as the ratio of the number of virus particles to the number of target cells present in these cultures, was 0.1.

Therefore, in our research the concentration of fluorescent nanospheres (viral phantoms) with a diameter of 100 nm was assumed at the level of approx. $24 \cdot 10^{12}$ particles per 3 mL, corresponding to the viral titer after incubation of the infected cell culture, e.g. Calu3 - human lung cancer cell line, cells over 5 days [*Chu et al., 2020*] with a 1000-fold allowance ensuring the possibility of optical evaluation of nanoparticle transmission in the ventilator tract.

Technical studies of the transmission of nanoparticles/solutions in two respiratory branches of the Ventil system were carried out in the period April 4 - 29, 2020.

1. Experimental

Chemicals and materials

Fluorescent compounds: sodium fluorescein (fluorescein) and methylene blue

- Virus phantom: fluorescent red and green polystyrene nanospheres (NS) with a diameter of 100 nm. The stable fluorescence of the nanospheres was ensured by the incorporation of a dye inside them (so-called internal marking). Suspension density: 1.05 g/cm^3.
  - Red Fluorescent Polystyrene Microspheres EPRUI-RF-100C
  https://www.epruibiotech.com/product/red-fluorescent-polystyrene-microspheres/
  Excitation / Emission Wavelength**:** 535 nm / 610 nm
  - Green Fluorescent Polystyrene Microspheres EPRUI-GF-100C
  https://www.epruibiotech.com/product/green-fluorescent-polystyrene-microspheres/
  Excitation / Emission Wavelength: 488 nm / 525 nm
  Refractive Index: 1.59@589nm(25℃)

Testing solutions and suspensions

Fluorescein and methylene blue solutions with a concentration of 1 mM were used in the research. In contrast, fluorescent nanospheres were administered as a 60 µL suspension and 240 µL stock suspension (provided by the manufacturer) per 3 mL of deionized water. The estimated number of nanospheres in the above-mentioned the suspensions were $6.5 \cdot 10^{12}$ and $24 \cdot 10^{12}$ particles/3 mL.

Experimental measurement system/setup

Figure R1 shows a diagram of the test set-up with the Ventil apparatus supplying the respiratory tract during independent ventilation of two artificial lungs (R and L). The measuring stand was prepared by the Ventil project team. Information on the settings of the ventilator and the associated Ventil is provided in Appendix 1. The L: R flow ratio was 6:4. In order to test the possibility of transmission between the airways, the test solutions/suspensions were administered to the expiratory tract of the artificial L lung using the Areogen nebulizer (N) in 3 mL portions repeated several times. The nebulizer operated in the mode of continuous and intensive nebulization - consumption of 5 mL solution within 30 minutes.

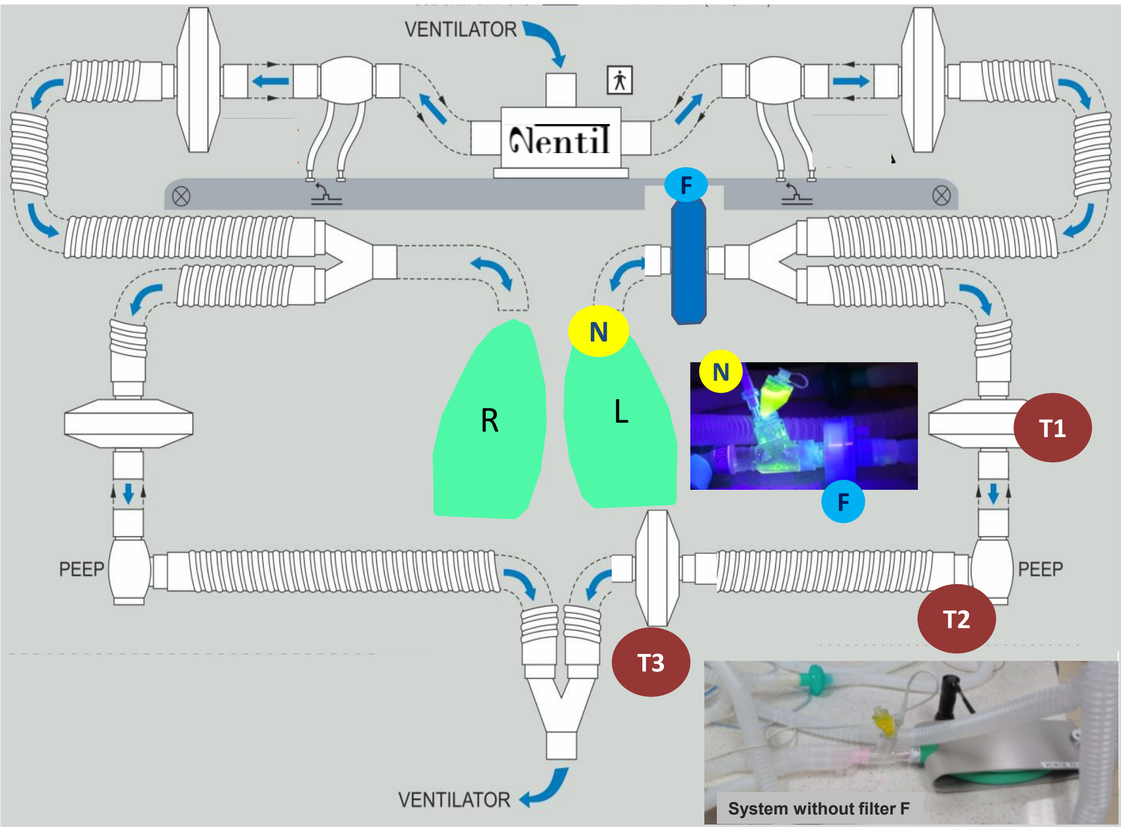

Fig. R1. Diagram of the test measurement system with the Ventil apparatus in the respiratory tract during independent ventilation of two artificial lungs. F- electrostatic hydrophobic filter, N- nebulizer, T1, T2 and T3 - test points. The ratio of the flows in the respiratory paths L:R = 6:4.

The presence of test substances and nanoparticles was examined at test points T1, T2, and T3. Fluorescence observations of the respiratory tract elements, including filters, were carried out and documented with photos taken with a digital camera. The highlights of the respiratory tract were made with the use of a diode emitting UV radiation with a wavelength in the range of 360 nm.

Due to the fact that in the UV light autofluorescence of materials from which the elements of the measurement path are made is easily visible as a pink glow, attention should be paid to the selection of the colors of fluorescent substances used in the tests. After the pre-testing of visibility of the fluorescent dyes and nanospheres in the system, fluorescein and red fluorescent nanospheres were selected for the further study.

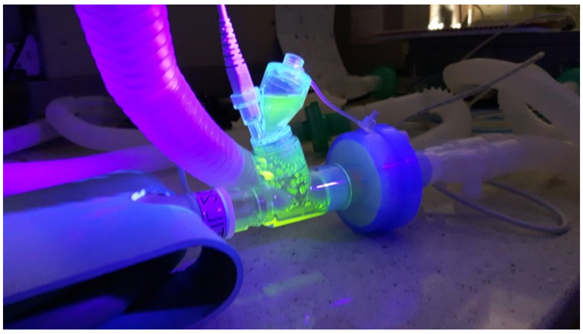

Fig. R2. Typical position of the electrostatic filter F in the patient's respiratory tract during respiration. In this case, with the attached nebulizer containing a fluorescein solution.

In the case of using the electrostatic filter F in the position shown in Fig R1 and Fig. R2, typically used during artificial ventilation of the patient, no traces of fluorescent substances introduced into the system using a nebulizer were observed at the outlet of the F filter.

In the further part of the study, the F filter was applied from the artificial lung side upstream of the nebulizer or was not included in the system. Removal of filters from the system created favorable conditions for the transfer of test solutions and suspensions through the system.

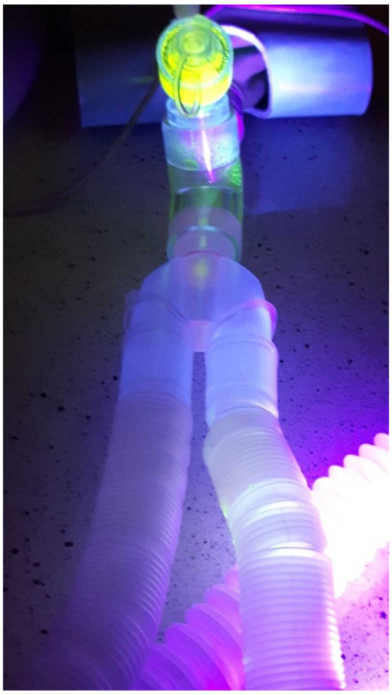

Fig. R3. System without F-filter during administration of fluorescein solution.

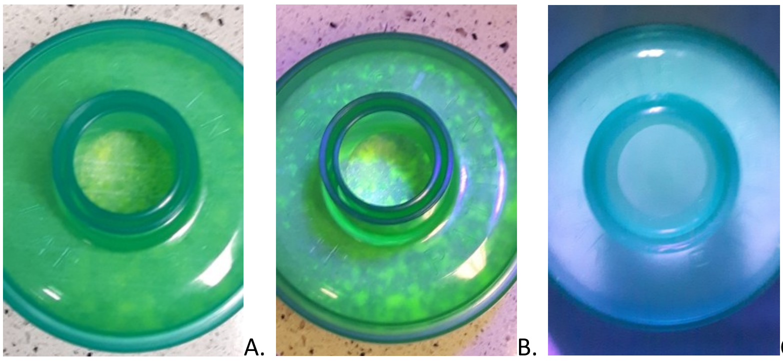

A. B. C.

Fig. R4. Filters from the test points: T1 (A), T2 after removing the PEEP (B) and T3 under UV light (C), after exposure to fluorescein solutions fed into the system using a nebulizer.

System without F electrostatic filter in the respiratory path with connected nebulizer containing fluorescein solution (Fig. R3). Fluorescein solution (yellowish green glow) is visible in the expiratory tract (right pipe). However, due to the overpressure in the inspiratory tract (left pipe), the fluorescein solution is not introduced there despite the lack of an F filter.

The respiratory tract filters were placed individually at the test points T1, T2, and T3. The surface of the filters at test points after exposure to four nebulization cycles of fluorescein solutions is shown in Figure R4. There were no traces of fluorescein on the post-filtering surfaces of the filters located in the test points. Furthermore, no traces of fluorescein were observed on the filtering surface of the filter placed at test point T3.

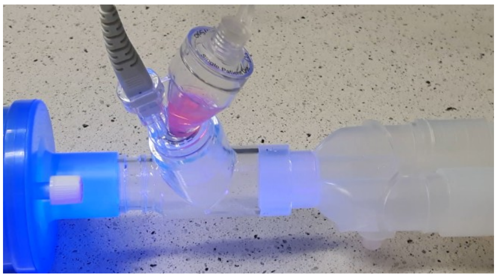

Fig. R5. System with F-filter placed on the side of the artificial lung when administred a suspension of red fluorescent nanospheres.

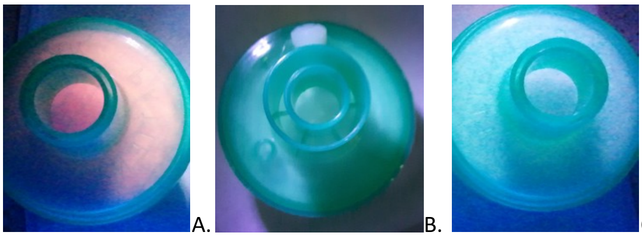

Fig. R6. Filter at test point T1, filtering surface (A) and post-filtering surface (B), and filtering surface of the filter at point T3 when the PEEP valve removed (C), after exposure to 4 nebulization cycles of a suspension of nanospheres containing a red fluorescent dye. Photographs under UV light.

After exposure to 4 nebulization cycles, traces of the suspension of fluorescent nanospheres were observed only on the filter surface of the filter placed at test point T1 (Fig. R6. A and B). At the T2 test point, traces of nanospheres were found on the filtration surface (no photo was included due to poor quality). However, at the last test point T3, no nanoparticles were found on the filtration surface.

2. Summary

The report presents the results of technical studies on the transmission of nanoparticles / solutions in two respiratory branches of the Ventil system. The experiment conditions were selected in such a way that they were unfavorable from the point of view of protection against transmission - introducing individual filters into the system at subsequent test points. Traces of fluorescein on the filtering surface of the filter at test point T3 (ventilator entry) only occur with fluorescein solution. On the other hand, in the case of fluorescent nanoparticles introduced from the suspension with an excess concentration (approx. 3 orders of magnitude), they appear on the filtering surface of the filter at point T1. On the basis of the conducted technical studies related to the assessment of the possibility of transmission of solutions/suspensions in two respiratory branches of the Ventil system, it was found that under the conditions of the experiment with the use of fluorescent solutions and suspensions no transmission occurs. To complement, based on the research it was found that there was no transmission of phantoms of virus - fluorescent nanospheres of 100 nm diameter, between the two pathways of the respiratory system during independent ventilation of two artificial lungs using the Ventil apparatus.

The independent studies described in this report, in some part related to the use of solutions of fluorescent compounds to evaluate the possibility of transmission, were conducted under similar conditions and are consistent with the results published in the article [*Srinivasan et al. 2020*]. In relation to the research described in the above-mentioned paper (continuous nebulization of a 5 mL solution of trypan blue for 10 minutes, and visual observation of filters), in our experiment, apart from use fluorescein and methylene blue solutions, we proposed a method of examination of transmission, which used phantoms of virus in the form of internally labeled with fluorescent dyes nanospheres with a diameter of 100 nm, the concentration of which in the administered suspension was approx. $24 \cdot 10^{12}$ particles/3 mL, corresponding to the viral titer with ca. 1000-fold excess, in respect to SARS-CoV-2 titer after incubation of the infected Calu3 cell culture for more than 5 days [*Chu et al., 2020*]. Fluorescent solutions and suspensions were introduced into the respiratory tract in 4 continuous nebulization cycles lasting 30 minutes each. Research with the use of nanoparticles can be carried out in laboratory environment, giving preliminary information about the possibility of cross-transmission in the respiratory tract during independent ventilation of artificial lungs.

3. References

Chu H, Chan JF-W, Yuen TT-T, Shuai H, Yuan S, Wang Y, et al., (2020). *Comparative tropism, replication kinetics, and cell damage profiling of SARS-CoV-2 and SARS-CoV with implications for clinical manifestations,*

transmissibility, and laboratory studies of COVID-19: an observational study. The Lancet Microbe. doi:10.1016/s2666-5247(20)30004-5
Fabian P, McDevitt JJ, DeHaan WH, Fung ROP, Cowling BJ, et al. Influenza Virus in Human Exhaled Breath: An Observational Study. PLoS ONE 2008, 3(7): e2691. doi: 10.1371/journal.pone.0002691.
Srinivasan S, Ramadi KB, Vicario F, Gwynne D, Hayward A, Lagier D, et al., (2020). *A rapidly deployable individualized system for augmenting ventilator capacity. Science Translational Medicine, eabb9401.* doi:10.1126/scitranslmed.abb9401

Appendix 1. Information on the settings of the ventilator and the associated Ventil device

Ventil project team:

Dr inż. Krzysztof Zieliński – leader of the project
Prof. dr hab. inż. Marek Darowski
Mgr inż. Anna Stecka
Dr inż. Marcin Michnikowski
Mgr inż. Piotr Okrzeja
Dr inż. Maciej Kozarski
Dr inż. Katarzyna Kramek-Romanowska
Dr inż. Krzysztof Jakub Pałko
Dr hab. inż. Piotr Ładyżyński – coordinator of the project

The measuring stand for studying the transmission of nanoparticles/solutions in two respiratory branches of the Ventil system was prepared by the Ventil project team. Information on settings in the ventilator system and the associated Ventil device is presented below.

Volume Controlled (VC) Mode

- Respiratory rate, fb = 9.1 [1/min]
- Set tidal volume, TV = 600 [mL]
- End-inspiratory pause, Tpl = 0.0 [s]
- Inspiratory flow shape - rectangular
- Maximum flow rate (for a rectangular signal equal to the flow during inspiration),
- $\dot{V}$ = 22 [L/min]
- PEEP, PEEPe valve setting = 0.0 [cm H_2O]
- Oxygen fraction, FiO_2 = 0.21 (in the experimental conditions, the setting is irrelevant due to the supply of air to the oxygen input)
- Trigger for flow, $\dot{V}$ = 3 L/min

Ventilator readouts:

- Peak pressure, P_{peak} = 16 cm H_2O
- Medium pressure, P_{mean} = 6.7 cm H_2O
- Internal PEEP, $PEEP_i$ = 4 cm H_2O
